# Cholesterol and ORP1L-mediated ER contact sites control autophagosome transport and fusion with the endocytic pathway

**DOI:** 10.1038/ncomms11808

**Published:** 2016-06-10

**Authors:** Ruud H. Wijdeven, Hans Janssen, Leila Nahidiazar, Lennert Janssen, Kees Jalink, Ilana Berlin, Jacques Neefjes

**Affiliations:** 1 Division of Cell Biology, The Netherlands Cancer Institute, Plesmanlaan 121, 1066 CX Amsterdam, The Netherlands; 2 Department of Chemical Immunology, Leiden University Medical Center, 2300 RC Leiden, The Netherlands

## Abstract

Autophagy is the main homeostatic pathway guiding cytosolic materials for degradation by the lysosome. Maturation of autophagosomes requires their transport towards the perinuclear region of the cell, with key factors underlying both processes still poorly understood. Here we show that transport and positioning of late autophagosomes depends on cholesterol by way of the cholesterol-sensing Rab7 effector ORP1L. ORP1L localizes to late autophagosomes and—under low-cholesterol conditions—contacts the ER protein VAP-A, forming ER-autophagosome contact sites, which prevent minus-end transport by the Rab7–RILP–dynein complex. ORP1L-mediated contact sites also inhibit localization of PLEKHM1 to Rab7. PLEKHM1, together with RILP, then recruits the homotypic fusion and vacuole protein-sorting (HOPS) complex for fusion of autophagosomes with late endosomes and lysosomes. Thus, ORP1L, via its liganding by lipids and the formation of contacts between autophagic vacuoles and the ER, governs the last steps in autophagy that lead to the lysosomal degradation of cytosolic material.

## Abstract (web summary)

Autophagy requires transport of autophagosomes to the perinuclear region. Here, the authors show that ORP1L localizes to autophagosomes and mediates formation of ER contact sites that prevent autophagosome transport and fusion with endocytic vesicles when cholesterol levels are low.

## Graphical abstract

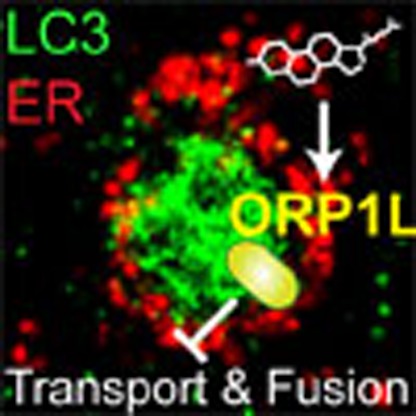

Autophagy is the primary pathway for degradation of cytosolic material, protein aggregates and damaged organelles in lysosomes (Lys), with its de-regulation implicated in neurodegenerative diseases, compromised immunity and cancer^1^,^2^,^3^. Cargo destined for such proteolysis becomes sequestered into double-membraned autophagosomes, lined with the distinguishing modifier LC3. Following membrane-closure these structures fuse with lysosomes, giving rise to a hybrid organelle—the autolysosome. Alternatively, autophagosomes progress through an intermediate amphisome stage—the result of fusion with late endosomes (LEs)—and subsequently mature by acquiring lysosomal characteristics or merge with the pre-existing lysosomes in a secondary fusion event^4^. While many of the essential molecular players for these processes have been identified, our understanding of the underlying factors controlling transport and fusion of autophagic vacuoles (AVs, which include autophagosomes, amphisomes and autolysosomes) remains incomplete.

Entry of autophagic cargoes into the endocytic system is orchestrated by components from both the autophagosome and the late endosomal/lysosomal (LE/Ly) side. A complex consisting of autophagosome-localized ATG14L, the SNAREs STX17 and SNAP29 as well as endosomal VAMP8 has recently been shown to execute the heterologous autophagosome/LE (or autophagosome/Ly) fusion event^5^,^6^,^7^,^8^. Tethering of the participating membranes, which precedes fusion, critically depends on both STX17 (ref. 6) and the GTPase Rab7 (refs 9, 10, 11, 12), with the latter likely provided by the endosome. STX17 has been shown to recruit the homotypic fusion and vacuole protein-sorting (HOPS) complex, which is thought to bridge the two organelles, thus facilitating their unification by SNARE proteins^13^,^14^,^15^,^16^. In addition, PLEKHM1 as well as RILP—both effectors of Rab7—can bind HOPS^15^,^17^,^18^,^19^,^20^, with PLEKHM1 reportedly involved in autophagosome-LE/Ly fusion^17^. The above tethering and fusion proteins are recruited to the autophagosome/LE (or Ly) interface in a stimulus-dependent manner^14^,^17^, suggesting existence of additional factors to influence and fine-tune the fusion process.

Entry of autophagosomes into the late endosomal/lysosomal pathway requires dynein motor-mediated transport towards the microtubule organizing center^21^,^22^,^23^,^24^,^25^,^26^, where most lysosomes reside. However, the nature of motor recruitment control and its effects on transport of autophagosomes is largely unknown. During maturation, autophagosomes acquire Rab7, typically relegated to the LE/Ly^9^,^10^,^27^, perhaps as a result of fusion with these compartments. On endosomes, Rab7 can associate with several effectors, including RILP, which recruits the dynein–dynactin motor for minus-end transport^28^,^29^. Whether autophagosomes utilize this Rab7-associated transport and fusion machinery en route to the lysosome, and if so, what factors control this process, is not known.

Here we show that transport of autophagosomes is governed by two Rab7 effectors—RILP and the cholesterol-sensor ORP1L. ORP1L localizes to amphisomes and autolysosomes and, under low cholesterol conditions, contacts the ER protein VAP-A to form intimate membrane contact sites (MCSs) with the ER. These ER-autophagosome MCSs prevent dynactin binding to RILP, thus blocking dynein-mediated transport. Furthermore, ORP1L in its low-cholesterol state acts as a negative regulator of AV/LE fusion and prevents binding of both PLEKHM1 and HOPS by Rab7–RILP. Thus, our study reveals a molecular mechanism whereby ORP1L, via its liganding by lipids and the formation of contacts between AVs and the ER, controls the degradation of cytosolic components by autophagy.

## Results

### Cholesterol controls autophagosome positioning

**Figure 1:**
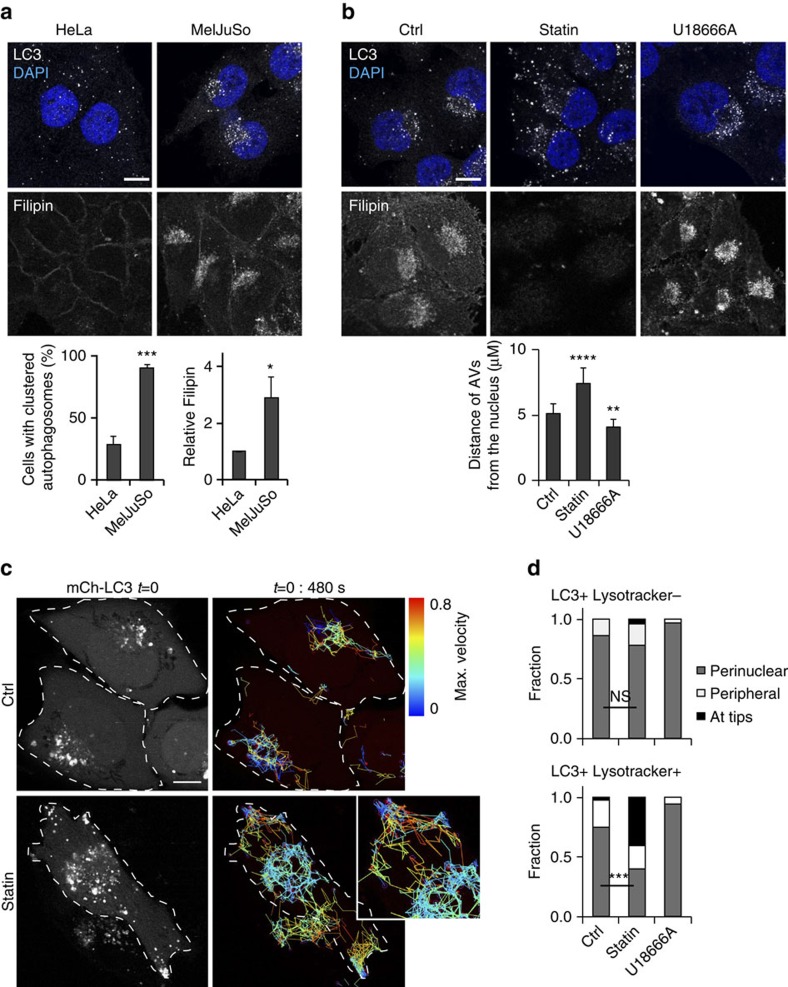
Cholesterol modulates autophagosome positioning. (**a**) HeLa or MelJuSo cells were stained for LC3 and 4,6-diamidino-2-phenylindole (DAPI), or with filipin to detect cholesterol. Scale bar, 10 μm. Panels below: quantification for the presence of a detectable perinuclear LC3 cluster: >100 cells were analysed per experiment. For filipin labelling, signal intensity was measured for at least three fields per experiment and was normalized to signal intensity in HeLa cells, which was set at 1. Bars indicate mean+s.d. from independent triplicates, significance calculated using Student's *t*-test. (**b**) MelJuSo cells cultured for 24 h under cholesterol-depleting conditions (see Methods) or exposed for 24 h to 3 μM U18666 were stained for LC3 and DAPI. Scale bar, 10 μm. Lower panel: quantification of the average distance (μm) of the AVs from the nucleus (using the LasAF software), from >10 cells per experiment. Bars indicate mean+s.d. from independent triplicates, significance calculated using Student's *t*-test. (**c**) Distribution and dynamics of mCherry-LC3-marked vesicles in control versus lipid-depleted MelJuSo cells. Confocal image at start of time lapse is shown on the left, vesicle trajectories over a 480-s interval are displayed on the right. Colours of individual vesicle trajectories reflect maximal displacement rate (blue=0, red=0.8 μm s^−1^) achieved during the 480-s interval. Scale bar, 10 μm. (**d**) Quantification of the localization of mCherry-LC3 vesicles either or not labelling for Lysotracker in MelJuSo cells treated as indicated. At least five cells per replicate were quantified, bars indicate mean+s.d. from independent triplicates. Significance calculated using Student's *t*-test (Supplementary Movies 1–3). NS, not significant, *P<0.05, ^**^P<0.01, ^***^P<0.001, ^****^P<0.0001). AV, autophagic vacuole.

Autophagic vacuoles are dynamic compartments that move bi-directionally along microtubules^20^,^21^,^28^ and mostly encounter lysosomes in the perinuclear region^30^. Intriguingly, steady-state cellular distribution of AVs varies greatly between different cell types. While in cervical HeLa cells AVs are dispersed throughout the cytoplasm, in melanoma MelJuSo cells most structures containing the autophagy marker LC3 localize at the perinuclear region (Fig. 1a). Since intracellular AV position is determined by the activities of kinesin and dynein motor proteins^21^,^22^,^31^ common to all cells, these differences might arise from variations in motor activity or recruitment. Strikingly, MelJuSo cells also contain more (endosomal) cholesterol (Fig. 1a), known to promote localization of endosomes and lysosomes at the microtubule minus-end^32^,^33^, close to the nucleus, raising the possibility that AV transport is modulated by endosomal cholesterol levels.

To test this, we reduced cholesterol levels in cells using statin (to inhibit endogenous cholesterol production, supplemented with mevalonate to provide essential nonsteroidal isoprenoids) and lipid-free serum (to inhibit the major source of cholesterol for cells as taken up through the LDL receptor)^34^. In addition, we accumulated cholesterol in the endosomal compartment by exposure of cells to U18666A (refs 32, 34), an inhibitor of the endosomal cholesterol exporter NPC1 (ref. 35). Strikingly, in the case of lipid depletion, AVs were found to scatter throughout the cytoplasmic space and accumulate at the tips of the cell, while they concentrated at the perinuclear region under conditions resulting in accumulated endosomal cholesterol. This was observed both in MelJuSo and HeLa cells, as well as for statin and lipid-free serum separately, ruling out non-cholesterol-mediated effects caused by statin (Fig. 1b; Supplementary Fig. 1a,b). To follow the dynamics of these AVs, we performed live-cell imaging using mCherry-LC3 and Lysotracker to label AVs and the late endocytic compartments. This indicated that the AVs localizing to the tips under low-cholesterol conditions were relatively static and remained located at their position for prolonged periods of time (Fig. 1c; Supplementary Movies 1–3), suggesting a defect in microtubule-based minus-end transport. Furthermore, the majority of these peripheral AVs labelled positive for Lysotracker (Fig. 1d and Supplementary Movies 1–3), as well as for Rab7 (Supplementary Fig. 1c), defining them as late or mature autophagosomes (amphisomes and autolysosomes). Thus, manipulating cholesterol affects the transport dynamics of late autophagosomes.

### ORP1L senses cholesterol to regulate autophagosome transport

**Figure 2:**
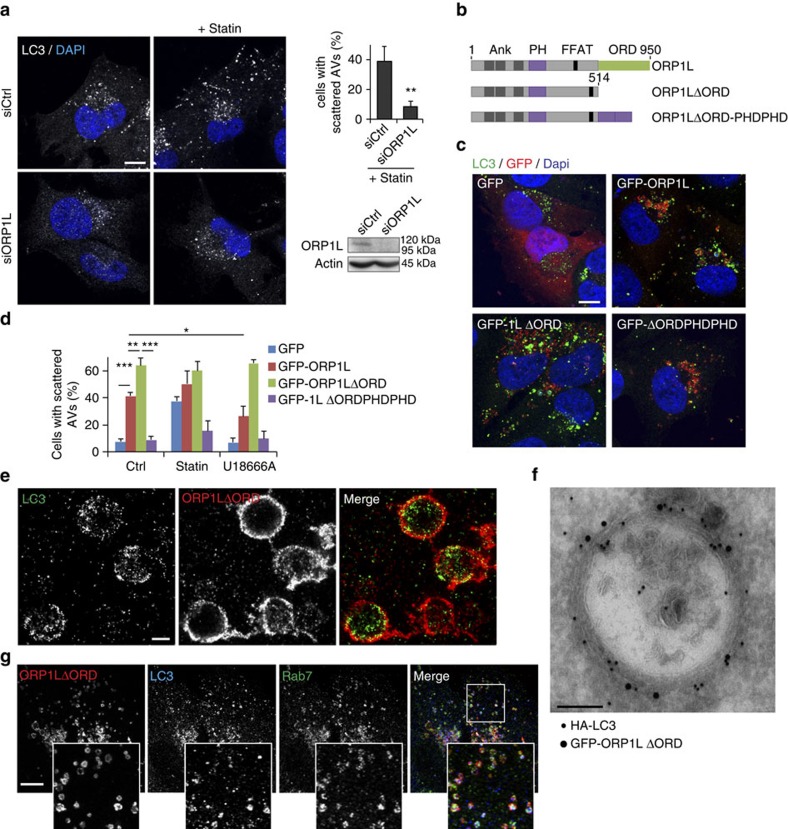
ORP1L senses cholesterol to control autophagosome positioning. (**a**) MelJuSo cells transfected with siCtrl or siORP1L were either or not cultured under lipid-depleted conditions and stained for LC3 and DAPI. Scale bar, 10 μm. Top right: quantification of scattering of LC3 structures under lipid-depleted conditions, over >100 cells were counted per experiment, bars indicate mean+s.d. from three experiments. Means were compared using Student's *t*-test. Lower right: western blot to validate silencing of ORP1L. (**b**) Overview of the ORP1L constructs and their domains used in this study. The C-terminal ORD domain binds oxysterols. (**c**) MelJuSo cells transfected with the indicated GFP-tagged constructs were fixed and stained for LC3 and DAPI. Scale bar, 10 μm. (**d**) Quantification of cells with scattered autophagic vesicles in cells under different cholesterol manipulation conditions and expressing different GFP-tagged constructs, as shown in **d** and Supplementary Fig. 2b. Quantification was performed on >50 transfected cells per experiment, bars indicate mean+s.d. from independent triplicates. Significance calculated using Student's *t*-test. (**e**) Superresolution microscopy on cells expressing HA-ORP1LΔORD, stained for HA and LC3, as indicated. Scale bar, 500 nm (**f**) Cryo-immuno-EM on HeLa cells expressing HA-LC3 and GFP-ORP1LΔORD stained with HA^10 nm^ and GFP^15 nm^ gold antibodies, respectively. Scale bar, 100 nm. (**g**) MelJuSo cells expressing GFP-ORP1LΔORD were fixed and stained for Rab7 and LC3. Scale bar, 10 μm. *P<0.05, ^**^P<0.01, ^***^P<0.001. AV, autophagic vacuole.

A key distinguishing characteristic between early and late AVs is the presence of Rab7 (ref. 36), suggesting that Rab7 effectors could be responsible for cholesterol-dependent late autophagosome re-localization. We, and others, have previously shown that the Rab7 effector ORP1L acts as a cholesterol sensor on late endosomes^33^,^37^. Upon (membrane) cholesterol binding by its ORD domain, ORP1L maintains a conformation compatible with dynein motor recruitment and minus-end transport, while absence of cholesterol prevents this^33^,^37^. Silencing ORP1L in MelJuSo cells indeed reduced the fraction of AVs localizing at the cell periphery under basal conditions (Fig. 2a; Supplementary Fig. 2a). Furthermore, ORP1L knockdown strongly inhibited AV scattering caused by cholesterol depletion (Fig. 2a), illustrating a critical role for ORP1L in cholesterol-mediated AV repositioning. To test whether cholesterol controls positioning directly via ORP1L, we expressed GFP-tagged wild-type ORP1L or variants altering its cholesterol-sensing properties (Fig. 2b). These were: ΔORD, lacking the cholesterol-interacting ORD domain (thereby mimicking the non-cholesterol bound state of ORP1L and acting as a cholesterol-independent inhibitor of dynein recruitment, since this variant cannot be inactivated by cholesterol) and ΔORDPHDPHD, where the ORD domain is replaced by a tandem membrane-targeting PH domain (PHD) to reflect the membrane-bound high cholesterol state (acting as a cholesterol-independent permissive factor for dynein binding, since the tandem PHD domains remain membrane bound and allow dynein recruitment, mimicking the high cholesterol state even under conditions of low cholesterol)^33^ (modelled in Supplementary Fig. 2b). These constructs mimic the low or high late endosomal cholesterol states, respectively, independently of the actual cholesterol concentration, since the ORD domain has been removed^33^. Expression of ORP1L and especially ORP1L–ΔORD, but not ORP1L–ΔORDPHDPHD, resulted in profound scattering of AVs (Fig. 2c,d), indicating that ORP1L regulates AV positioning as a function of its cholesterol-bound state. Importantly, cholesterol depletion induced scattering of AVs in cells ectopically expressing GFP, but had no effect on cells expressing ORP1L–ΔORDPHDPHD, implying that low cholesterol levels had the effect through the ORP1L protein (Fig. 2d and Supplementary Fig. 2c). In line with this, GFP-ORP1L and even more pronounced the ORP1LΔORD construct resisted AV clustering following cholesterol accumulation by U18666A, unlike GFP or the ORP1LΔORDPHDPHD construct mimicking the high-cholesterol form of ORP1L (Fig. 2d; Supplementary Fig. 2c). While cholesterol manipulation of cells may result in several effects on lipids and proteins, the fact that the low- and high-cholesterol state configurations of ORP1L override these effects suggests that the effects of cholesterol on AV positioning are largely ORP1L mediated.

To investigate which AV substructures are subject to regulation by ORP1L, we investigated the types of structures ORP1L localized to. As assessed by super-resolution total internal reflection (TIRF) microscopy, ORP1L localized to the limiting membrane of AVs, (Fig. 2e), which was confirmed by cryo-immuno-EM, where ORP1L was found to localize to single-membrane AVs, which defines them as amphisomes or autolysosomes (Fig. 2f). In support of this, all ORP1L marked AVs co-labelled for Rab7 (Fig. 2g; Supplementary Fig. 2d). Together, these data suggest that cholesterol as sensed by ORP1L dictates the intracellular position of Rab7-marked late autophagosomes.

### Low cholesterol and ORP1L yield of ER-AV contact sites

**Figure 3:**
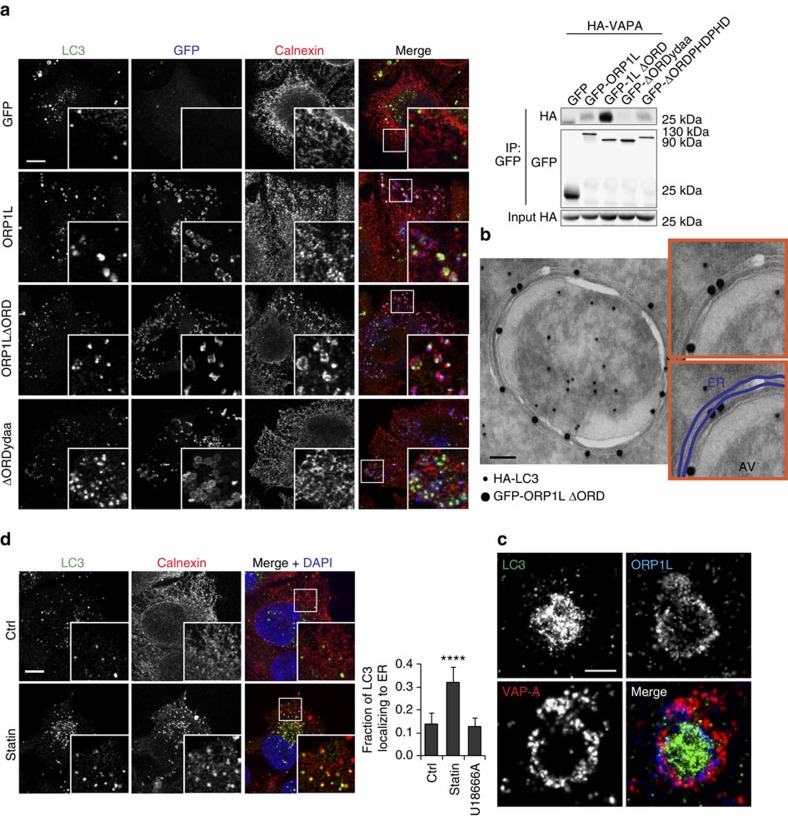
ORP1L and VAP-A form cholesterol-dependent ER-AV contact sites (**a**) MelJuSo cells expressing GFP or GFP-tagged ORP1L mutants were fixed and stained for LC3 and Calnexin. Y477 and D478 in the FFAT motif were mutated to alanines (A) in the ORP1L ydaa mutant. Scale bar, 10 μm. Right: co-immunoprecipitation for ORP1L (mutants) with VAP-A. GFP-ORP1L mutants or GFP were isolated from lysates of HEK293T cells co-overexpressing HA-VAPA using GFP-Trap beads. Western blot filters were probed for isolated GFP-tagged proteins, the associated HA-VAP-A and the input HA-VAP-A, as indicated. (**b**) Cryo-immuno-EM on HeLa cells expressing HA-LC3 and GFP-ORP1LΔORD, as detected by HA^10 nm^ and GFP^15 nm^ gold antibodies. Insets show ORP1L labelling in the membrane contact site between ER and autophagosome. The membranes of the ER are depicted in the bottom inset. Scale bar, 50 nm. (**c**) Three-colour super-resolution image of an autophagosomal vesicle labelled by LC3 (green), ORP1L (blue) and the ER protein VAP-A (red). Scale bar, 500 nm (**d**) MelJuSo cells cultured either in lipid depleted serum or control medium were fixed and stained for LC3 and ER marker Calnexin. Scale bar, 10 μm. Right panel: Manders coefficient for LC3 localization to the ER was calculated on at least 10 cells over three independent experiments. Bars indicate mean+s.d. Student's *t*-test statistical analysis (^****^P<0.0001).

When recruited to LE by Rab7, ORP1L has a conformational state allowing interactions with ER-protein VAP-A to establish a MCS between these two compartments. VAP-A then controls the binding of the dynein motor to RILP^33^,^38^. This interaction with VAP-A is stabilized by mutating or deleting the ORD domain of ORP1L and is promoted by low cholesterol culture conditions^38^ (Fig. 3a). To assess whether ORP1L has the ability to form physical contacts between late autophagosomes and the ER, coincidence of ER marker calnexin and LC3 was detected as a function of ORP1L mutants. Robust distribution of (calnexin positive) ER pockets to LC3^+^ structures occurred in the presence of either ORP1L or ORP1LΔORD, and could be prevented by eliminating the VAP-A-binding site FFAT (two phenylalanines (FF) in an Acidic Tract) in the latter (by mutating Y477 and D478 into alanines, Fig. 3a). Typical MCSs are characterized by a spatial separation of less than 10−30 nm between two compartments^39^, which required analyses of these contact sites by EM and superresolution light microscopy. Cryo-immuno-EM showed long stretches of ER closely positioned to vesicles positive for both ORP1L and LC3 (Fig. 3b; Supplementary Fig. 3a). 3-Colour superresolution TIRF microscopy confirmed the existence of VAP-A–ORP1L contacts on LC3 positive structures, with AVs containing ORP1L significantly surrounded by ER protein VAP-A (Fig. 3c). At this level of resolution, ORP1L was again found at the limiting membrane and surrounded by VAP-A, covering major parts of the vesicle (Supplementary Movie 4).

Similar to overexpression of ORP1L and ORP1LΔORD, cholesterol depletion profoundly stimulated re-distribution of the ER towards LC3 puncta, creating a distinct punctate ER contacting the LC3^+^ structures (Fig. 3d; Supplementary Fig. 3b). Notably, silencing of either ORP1L or VAP-A reduced these ER contacts with LC3 in the cell periphery, while perinuclear MCSs were only sensitive to silencing VAP-A (Supplementary Fig. 3c). This suggests that cholesterol controls the formation of ORP1L–VAP-A contact sites, as well as contact sites between VAP-A and a different autophagosomal protein. Together, these results visualized a novel membrane contact site, one between late autophagosomes and the ER.

### RILP regulates transport of late autophagosomes

**Figure 4:**
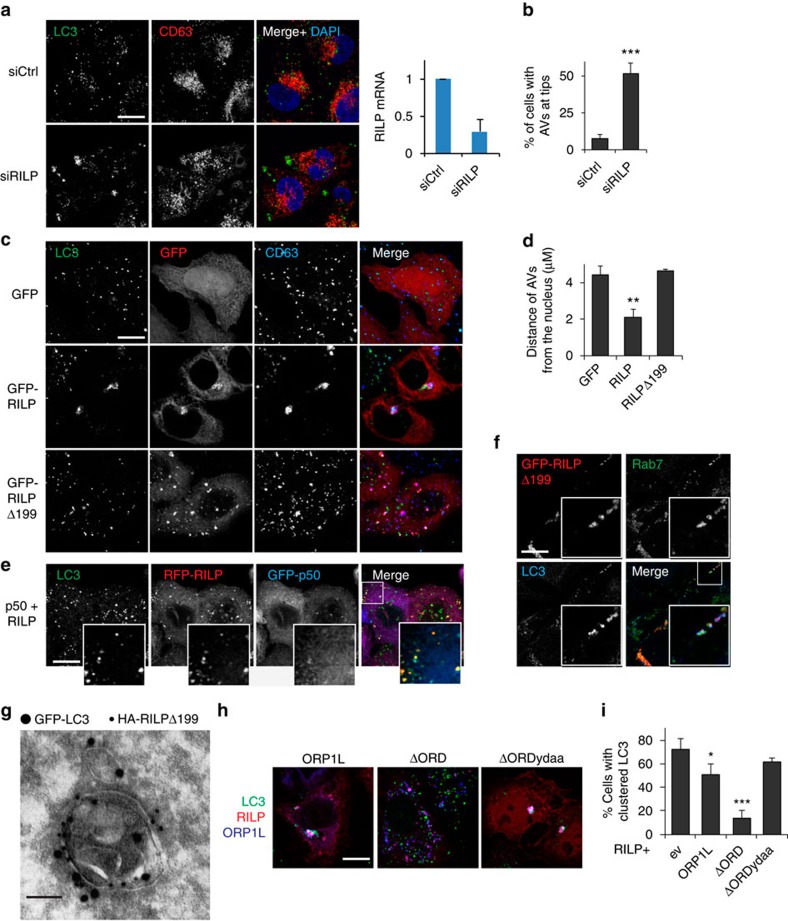
ORP1L controls RILP mediated transport of late autophagosomes. (**a**) MelJuSo cells transfected with siCtrl or siRILP were stained for LC3, CD63 and DAPI. Scale bar, 10 μm. Right panel; silencing efficiency of RILP was determined by qRT-PCR, RILP signal was normalized to GAPDH. Bars indicate mean+s.d. of three experiments. (**b**) Quantification of cells with AVs located at the tips of cells from **a**, >100 cells per experiment were quantified, bars show mean+s.d. of three independent replicates. Means were compared using Student's *t*-test (**c**) HeLa cells transfected with the indicated constructs were fixed and stained for LC3 and CD63, as indicated. Scale bar, 10 μm. (**d**) Quantification of the average distance (μm) of AVs from the nucleus (by LasAF software) as shown in **c**, from >10 cells per experiment. Bars display mean+s.d. of three independent experiments, significance was calculated using Student's *t*-test. (**e**) HeLa cells transfected with the indicated constructs were stained for LC3. Scale bar, 10 μm (**f**) MelJuSo cells expressing GFP-RILPΔ199 were stained for Rab7 and LC3. Scale bar, 10 μm. (**g**) Immuno-electron micrograph of a HeLa cell expressing GFP-LC3 and HA-RILPΔ199, detected with 15 and 10 nm gold, respectively. Note the single membrane around the LC3-positive structure. Scale bar, 100 nm (**h**) HeLa cells expressing GFP-RILP and HA-tagged ORP1L constructs were fixed and stained for HA and LC3. Scale bar, 10 μm. (**i**) Quantification of the number of cells with perinuclear accumulation of LC3-positive structures from **h**. Quantified >100 cells per experiment. Bars indicate mean+s.d. from independent triplicate experiments. Student's *t*-test statistical analysis. *P<0.05, ^**^P<0.01, ^***^P<0.001. ev, empty vector control.

On LE/Ly, ORP1L forms a tripartite complex with Rab7 and RILP and controls binding of the dynein motor complex to RILP^33^,^40^. RILP is also found on AVs and its recruitment to Rab7 is enhanced under conditions that promote the autophagic flux^41^, suggesting that RILP could also drive dynein acquisition on late autophagosomes—and is by extension sensitive to ORP1L. To assess this, we silenced RILP in MelJuSo cells, which yielded AV scattering and accumulation in the tips of the cell (Fig. 4a,b). Conversely, ectopic expression of RILP in HeLa cells produced strong clustering of AVs in the perinuclear region (Fig. 4c,d), while a RILP mutant deficient in dynein–dynactin interactions^40^ (Δ199) left the AVs scattered in the cell periphery. Furthermore, RILP induced recruitment of the dynein–dynactin subunit p150^glued^ to AVs (Supplementary Fig. 4a). RILP-mediated AV repositioning is dynein dependent, as disruption of the dynein motor complex by ectopically expressing p50^dynamitin^ (ref. 42), which by itself accumulated AVs at the tips of cells (Supplementary Fig. 4b), negated the perinuclear clustering induced by RILP (Fig. 4e). RILP localized to a fraction of the LC3^+^ vesicles (Fig. 4e), constituting the Rab7-marked late autophagosomal pool (Fig. 4f). This was further supported by immuno-EM analysis that showed RILP(Δ199) (full-length RILP strongly clustered AVs, LEs and Lys in the perinuclear region, thus preventing proper analyses) localizing to the limiting membrane of single membrane autophagosomes (amphisomes and autolysosomes^43^; Fig. 4g). These data suggest that the Rab7 effector RILP recruits dynein for minus-end transport of mature autophagosomes.

To assess the effect of ORP1L on RILP-mediated transport, we co-expressed RILP with ORP1L. While overexpression of ORP1L had only minor effects on RILP-induced perinuclear clustering of AVs (Fig. 4h,i), ORP1LΔORD completely eliminated this clustering, an effect dependent on its ability to bind VAP-A, since mutating the interacting FFAT motif in ORP1LΔORD restored RILP-induced perinuclear clustering. Similar to this, recruitment of dynactin subunit p150^glued^ to AVs was blocked by co-expression of RILP with ORP1LΔORD (Supplementary Fig. 4a). These data indicate that Rab7-effector RILP recruits the dynein motor to late autophagosomes for minus-end transport, and that ORP1L further controls this transport via the formation of ER-contact sites with VAP-A.

### ORP1L controls autophagosome maturation

**Figure 5:**
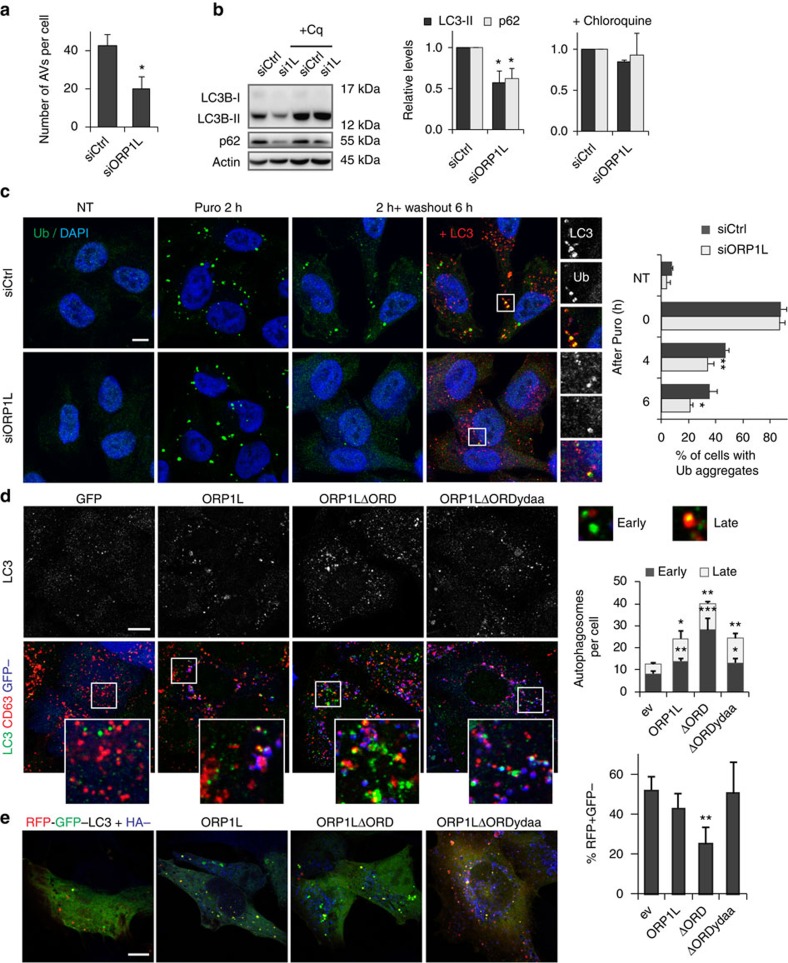
ORP1L controls autophagosome maturation. (**a**) Quantification of the number of autophagosomes in MelJuSo cells transfected with siCtrl or siORP1L, from Fig. 2a. Number of LC3 structures was quantified in >10 cells per experiment, bars show mean+s.d. from three independent replicates. Means were compared using Student's *t*-test. (**b**) MelJuSo cells transfected with siCtrl or siORP1L were either or not exposed to 50 μM chloroquine for 4 h, lysed and analysed by SDS–PAGE and western blotting for p62, LC3B and actin. Right panels, quantification of signals for p62 and LC3B-II, corrected for loading (actin) and normalized to siCtrl, untreated (left) and chloroquine treated (right). Bars correspond to mean+s.d. of three experiments, significance was calculated using Student's *t*-test. (**c**) MelJuSo cells transfected with siCtrl or siORP1L were left untreated (NT) or treated with 4 μg ml^−1^ puromycin for 2 h. Then, cells were washed and left to recover for the indicated time points before fixation and staining for ubiquitin (Ub), LC3 and DAPI. Scale bar, 10 μm. Right panel: quantification of percentage of cells with cytosolic Ub aggregates, >100 cells per experiment and bars represent mean+s.d. of three independent experiments. Means were compared using Student's *t*-test. (**d**) HeLa cells expressing the indicated GFP-tagged constructs were fixed and stained for LC3, CD63 and DAPI. Scale bar, 10 μm. Right panel: quantification of LC3+CD63− (early, green) and LC3+CD63+ (late, yellow) autophagosomes detected in cells with indicated expressed proteins. Quantification of at least 10 cells per experiment, bars represent mean+s.d. of three independent experiments. Significance calculated using Student's *t*-test. (**e**) MelJuSo cells expressing the indicated HA-tagged constructs in combination with the mRFP-GFP-LC3 tandem construct were starved for 4 h, fixed and stained for HA. Scale bar, 10 μm. Right panel: quantification of percentage of RFP-only LC3 structures relative to total LC3 labelled structures. Quantification of at least 10 cells per experiment is shown. Bars indicate mean+s.d. from independent triplicate experiments, statistical analysis using Student's *t*-test. *P<0.05, ^**^P<0.01, ^***^P<0.001. ev, empty vector.

Since transport of AVs is associated to their maturation, we reasoned that ORP1L might inhibit the autophagic flux. Consistent with this, ORP1L silencing reduced the number of LC3^+^ structures in cells (Figs 2a and 5a), as well as overall LC3B-II and p62 levels (Fig. 5b). This decrease was negated by blocking lysosomal degradation (Fig. 5b), illustrating that ORP1L depletion spurs the autophagic flux. Decreased p62 levels could imply that ORP1L also controls clearance of ubiquitinated protein aggregates. Aggregate-like induced structures are formed on cellular stress and recognized by selective autophagy receptors, including p62, for targeting to the autophagic pathway. While ORP1L knockdown had no effect on the generation of puromycin-induced aggregate-like induced structures, the number of cells containing ubiquitinated aggregates at 4 and 6 h following puromycin washout was reduced when compared with control cells (Fig. 5c). At these time points, most remaining aggregates stained positive for LC3, indicating effective targeting to the autophagosomal pathway. Thus, silencing of ORP1L increases the autophagic flux and the rate of protein aggregate degradation.

Conversely, ectopic expression of ORP1L increased the number of AVs in cells, which was more pronounced by expressing ORP1LΔORD (Fig. 5d). This phenotype was largely corrected for by mutating the VAP-A interacting FFAT motif in ORP1LΔORD. Co-staining with the LE/Ly marker CD63 revealed that overexpression of either ORP1L or ORP1LΔORD increased the numbers of both early (LC3^+^CD63^−^) and late (LC3^+^CD63^+^) autophagosomes. Surprisingly, the most significant difference was observed for the early (ORP1L negative) autophagosomes, suggesting that in addition to fine-tuning dynein motor-driven transport, ORP1L controls autophagosome fusion with the LE/Ly. To test this, we used the tandem mRFP-GFP-LC3B construct that contains a pH insensitive mRFP and a pH sensitive GFP molecule to distinguish early (GFP^+^RFP^+^) from late (GFP^−^RFP^+^) more acidic autophagosomes^12^. Following amino-acid starvation to accelerate the autophagic flux, cells expressing the low cholesterol mimic ORP1LΔORD harboured a significantly elevated fraction of early autophagosomes when compared with cells expressing wild-type ORP1L and vector control (Fig. 5e). Thus, ORP1L-induced ER contact sites are able to modulate autophagosome maturation.

We then tested whether cholesterol depletion or accumulation would also affect the autophagic flux. Whereas cholesterol-lowering conditions did not yield measurable changes in autophagic flux (Supplementary Fig. 5a,b), in agreement with others^44^,^45^, we found that increased lysosomal cholesterol levels prevented autophagosome-LE/Ly fusion (positioning them in very close proximity, Supplementary Fig. 5a,b). Since cholesterol reduction affects many cellular processes, it could be that compensatory mechanisms are in place, or that the cholesterol reduction is not strong enough to observe an effect on autophagic flux. Yet, the observed block in fusion with ORP1LΔORD suggests a role for endosomal cholesterol in autophagosome maturation.

### ORP1L controls PLEKHM1/HOPS acquisition by RAB7–RILP

**Figure 6:**
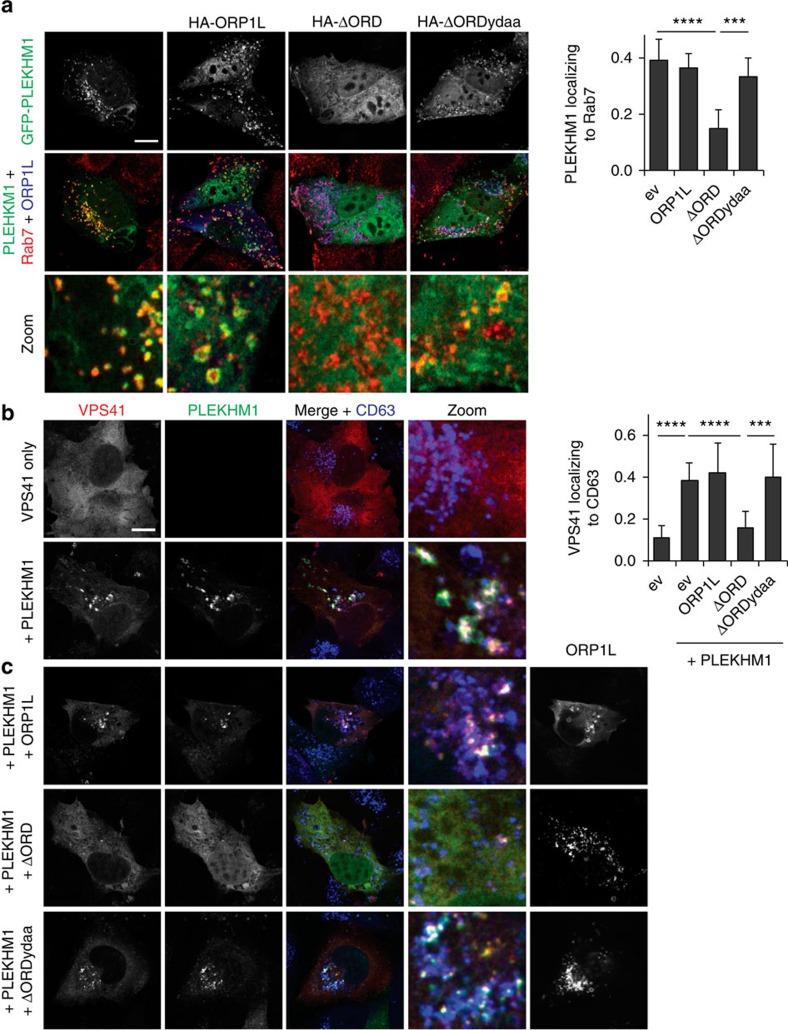
ORP1L regulates recruitment of PLEKHM1 and HOPS to Rab7 positive vesicles. (**a**) MelJuSo cells expressing GFP-tagged PLEKHM1 and the indicated HA-tagged ORP1L mutants were fixed and stained for Rab7. Scale bar, 10 μm. Right panel: Manders coefficient for PLEKHM1 localization to Rab7, as determined for >10 cells in three experiments. Bars represent mean+s.d. and significance calculated using Student's *t*-test. (**b**,**c**) MelJuSo cells were transfected with GFP-tagged PLEKHM1, mRFP-VPS41 and the indicated HA-tagged ORP1L mutants and stained for CD63. Manders coefficient for VPS41 localization to CD63 was determined for >10 cells in three experiments. Scale bar, 10μm. Bars display mean+s.d., means were compared using Student's *t*-test. ^***^P<0.001, ^****^P<0.0001. ev, empty vector.

The observations that ORP1L blocks autophagosome–LE/Ly fusion, together with the preferential localization of ORP1L to Rab7-positive compartments, suggest that ORP1L likely affects fusion between autophagosomes and LE/Lys from the latter side. Recent work has implicated the Rab7 effector PLEKHM1 as pertinent to this fusion step^17^. To test a possible functional interplay between ORP1L and PLEKHM1, we co-expressed GFP-tagged PLEKHM1 with ORP1L and its mutants. Strikingly, ectopic expression of ORP1LΔORD inhibited distribution of PLEKHM1 to Rab7, yielding cytosolic PLEKHM1 instead (Fig. 6a). Mutating the VAP-A interacting FFAT motif of ORP1L restored acquisition of PLEKHM1 by Rab7, suggesting that ORP1L does not simply compete with PLEKHM1 for Rab7-binding space, but rather actively informs PLEKHM1 recruitment in a manner dependent on the association with the ER protein VAP-A.

To mediate fusion, PLEKHM1 binds the HOPS complex, a multiprotein complex organizing the SNARE proteins for fusion^46^, likely through a direct interaction with HOPS subunit VPS39 (ref. 17). Whether this interaction drives recruitment of the entire HOPS complex to Rab7 is unknown. To test this, we expressed several HOPS subunits in combination with PLEKHM1. While all subunits tested were cytosolic when expressed on their own, co-expression of PLEKHM1 relocalized both VPS39 and VSPS41 to LEs/Lys (Fig. 6b; Supplementary Fig. 6a). No recruitment was observed for an alternate HOPS complex member, VPS33b (refs 16, 20; Supplementary Fig. 6b), implying that PLEKHM1 specifically recruits the HOPS core complex to Rab7. Introduction of ORP1LΔORD blocked membrane distribution of both VPS39 and VPS41, as well as PLEKHM1 in a manner dependent on the FFAT motif (Fig. 6c; Supplementary Fig. 6a). At the same time, ORP1LΔORD had no effect on localization of fusion factor VAMP8 (Supplementary Fig. 6c). These data support the premise that ORP1L-mediated ER-contact sites orchestrate tethering of autophagosomes with LE/Ly by modulating recruitment of the autophagic effector PLEKHM1, and subsequently that of HOPS, to Rab7.

**Figure 7:**
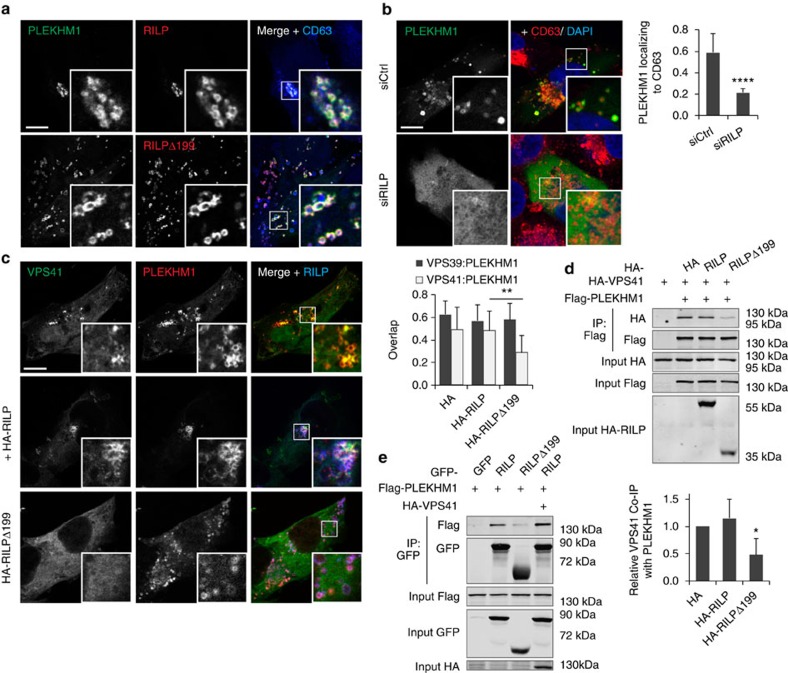
Rab7 effectors PLEKHM1 and RILP cooperate in HOPS complex recruitment. (**a**) MelJuSo cells were transfected with GFP-PLEKHM1 and HA-RILP or HA-RILPΔ199 and stained for CD63, as indicated. Scale bar, 10 μm. (**b**) MelJuSo cells silenced for siCtrl or siRilp were transfected with GFP-PLEKHM1 and stained for CD63. Manders coefficient for PLEKHM1 localization to CD63 was calculated for >10 cells over three experiments. Bars display mean+s.d., significance determined using Student's *t*-test. Scale bar, 10 μm. (**c**) MelJuSo cells were transfected with GFP-PLEKHM1 and mRFP-VPS41, together with HA-RILP or HA-RILPΔ199 when indicated. Manders coefficient for VPS39 (Supplementary Fig. 6c) or VPS41 localization to PLEKHM1 was calculated for >10 cells in three individual experiments. Bars represent mean+s.d. and significance was calculated using Student's *t*-test. Scale bar, 10 μm. (**d**) Co-immunoprecipitation for Flag-PLEKHM1 and HA-VPS41 in the presence of HA-RILP or HA-RILPΔ199. HEK293T cells were transfected with the indicated constructs and immunoprecipitated for Flag. Bottom: quantification of co-immunoprecipitated HA-VPS41 signal corrected for input and normalized to control, *n*=4. Bars display mean+s.d., means were compared using Student's *t*-test. (**e**) Immunoprecipitation for GFP or GFP-tagged RILP constructs in HEK293T cells co-expressing Flag-PLEKHM1 with or without HA-VPS41, as indicated. *P<0.05, ^**^P<0.01, ^***^P<0.001, ^****^P<0.0001.

**Figure 8:**
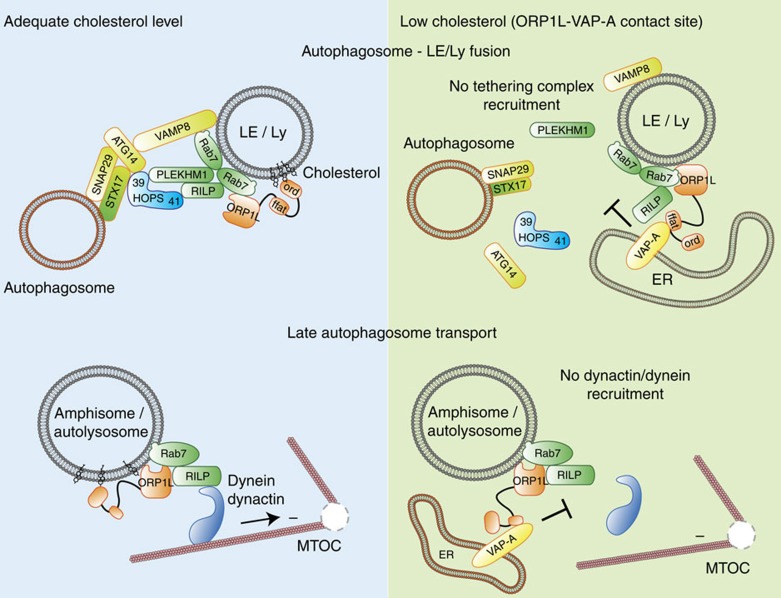
Model for ORP1L-mediated control of autophagosome–LE/Ly fusion and late autophagosome transport. SNARE proteins for fusion are depicted in yellow, tethering factors in green and the HOPS complex in blue. Tethering of autophagosomes with LE is orchestrated by two Rab7 molecules binding to two effectors PLEKHM1 and RILP, that respectively bind the VPS39 and VPS41 subunits of the HOPS tethering complex. From the autophagosome side, the SNARE Syntaxin 17 binds HOPS and mediates fusion together with the SNAREs SNAP29 and VAMP8, and ATG14. Under low-cholesterol conditions, the ORD domain of ORP1L is released from the membrane, exposing the FFAT motif that interacts with ER protein VAP-A. This membrane contact site prevents recruitment of PLEKHM1 and HOPS to Rab7–RILP, and thereby tethering and fusion. Once fusion does occur, RILP recruits dynactin–dynein to amphisomes to promote minus-end transport. Here ORP1L can also form a contact site with VAP-A, which prevents dynactin recruitment and blocks minus-end transport. Thus, conformational changes in the cholesterol-sensor ORP1L time the fusion and transport of AVs. LE, late endosome; Ly, lysosome; 39, VPS39; 41, VPS41.

Besides PLEKHM1, also RILP can recruit the HOPS complex to Rab7, through a direct interaction with VPS41 (refs 15, 19). This begs the question whether both Rab7 effectors—RILP and PLEKHM1—mediate fusion of different vesicular subpopulations, or rather cooperate to recruit HOPS, the latter possibility supported by the requirement for both adaptors in ensuring efficient access of endocytosed EGFR to the LE/Ly compartment^17^,^47^. RILP and PLEKHM1 robustly co-localized on the same vesicles (Fig. 7a; Supplementary Fig. 7a), and silencing RILP relegated PLEKHM1 to the cytosol (Fig. 7b), implying a functional interplay between the two effectors. The latter was not related to the HOPS recruitment capacity of RILP, since the RILPΔ199 mutant (which fails to recruit HOPS^15^) did not affect endosomal membrane targeting of PLEKHM1 (Fig. 7a; Supplementary Fig. 7a). Furthermore, expression of constitutively active Rab7(Q67L) restored membrane targeting of PLEKHM1 (Supplementary Fig. 7b), indicating that the phenotype is likely a result of an altered activated Rab7 state, as observed previously for effector silencing or overexpression^18^,^28^. While in the presence of RILPΔ199, PLEKHM1 was still able to recruit VPS39 (Fig. 7c; Supplementary Fig 7c), it poorly recruited VPS41 to endosomes (Fig. 7c). Co-immunoprecipitation experiments confirmed that the interaction between PLEKHM1 and VPS41 was attenuated by RILPΔ199 (Fig. 7d), implying that functional RILP is required for PLEKHM1 to mobilize VPS41 and thus the entire HOPS complex. RILP and PLEKHM1 physically associated with each other, which was strongly reduced by using RILPΔ199, and more efficient when co-expressing VPS41 (Fig. 7e), suggesting that RILP and PLEKHM1 form a complex via HOPS. Together, these observations suggest that the HOPS complex is jointly recruited to LE/Lys by the Rab7 effectors PLEKHM1 and RILP, where PLEKHM1 binds to HOPS subunit VPS39 and RILP to VPS41. Whereas RILP remains associated to Rab7 following formation of ORP1L–VAP-A contact sites by ORP1LΔORD^15^, PLEKHM1 and the HOPS complex are released, obstructing fusion of autophagosomes with LEs/Lys (Fig. 8).

## Discussion

Autophagic proteolysis of bulky cargoes acquired in the cytosol necessitates their entry into the endocytic system for degradation by lysosomal hydrolases. Endosomes present a challenging target, as they traverse different maturation stages, all while moving quickly between the peripheral and interior regions of the cell. How fusion and transport of autophagosomes are negotiated within this dynamic vesicle network in space and time is poorly understood. Here we show that maturing autophagosomes hijack late endosomal fusion and transport machinery to access the lysosomal compartment, and in doing so, become subject to endosome-centric regulatory mechanisms. Among these, we identify a negative regulator of autophagosome fusion with the LE/Ly—which functions as a cholesterol sensor poised on receiving vesicles to either licence or refute autophagic progression.

Enclosure of cytosolic substrates within LC3^+^ double-membranes gives rise to early autophagosomes, which mature by fusion with lysosomes or endosomes harbouring late characteristics. For fusion to take place, the autophagic membrane-bound SNARE protein STX17 (ref. 6) needs to contact the endosome-associated HOPS tethering complex^13^,^14^. On the LE/Ly side of the merger, the small GTPase Rab7 recruits its effectors PLEKHM1 and RILP, capable of respectively binding the VPS39 and VPS41 components of HOPS. Our data suggest that these effectors jointly recruit the same HOPS complex, which is analogous to yeast, where the Rab7 homologue YPT7 acquires HOPS by binding to both VPS39 and VPS41 (refs 48, 49). Since RILP and PLEKHM1 bind the same motif on Rab7 (ref. 18), two Rab7 molecules probably associate to the two different effector proteins—RILP and PLEKHM1—which associate to two subunits of one tethering HOPS complex.

In addition to PLEKHM1 and RILP, Rab7 recruits another effector, the cholesterol-sensor ORP1L, which binds Rab7 in the presence of RILP^40^ and engages directly with ER-located VAP-A. Genetic manipulation of underlying interaction determinants casts AV/LE fusion as subordinate to complex formation between ORP1L and VAP-A. In support of this, we find that in the presence of ORP1L predisposed to binding VAP-A, PLEKHM1 becomes relegated to the cytosol, while RILP remains associated to Rab7 but releases HOPS. This results in diminished entry of LC3^+^ compartments into the acidified luminal LE environment and expansion of the now-arrested early autophagosomal repertoire. Given that ORP1L-mediated ER-contact sites are modulated by cholesterol or oxysterols as recognized by the ORD domain of ORP1L, cholesterol on the cytosolic leaflet of Rab7–ORP1L compartments could control the autophagic flux. While the effects of the different ORP1L mutants on the autophagic flux revealed a clear and interpretable effect, this was less so for lipid manipulation of cells. This could be due to the milder nature of cholesterol manipulation, the multitude of other pathways that are affected by cholesterol, or another unknown factor controlling ORP1L contact site formation. However, our data with the ORP1L mutants suggest that cholesterol is instrumental for the autophagic flux, and demonstrate that the interplay between ORP1L and VAP-A determines whether a given AV/LE encounter will result in fusion, and by extension maturation of the autophagosome en route to proteolysis.

Following AV/LE fusion, the resulting amphisome needs to reach the lysosome to fulfil proteolysis of its contents. A large share of motor-based transport of autophagic vesicles is accounted for by amphisomes^21^,^24^,^50^, and several transport adaptors, including dynein intermediate chain-interacting proteins Snapin^24^,^51^ and Huntingtin^52^,^53^ are common between amphisomes and LEs. We demonstrate that LC3^+^ amphisomes retain both transport and regulatory determinants of its parent LE, all converging onto the small GTPase Rab7. On RILP engagement with amphisomes, the dynein–dynactin complex can be recruited for minus-end-directed transport towards lysosomes, typically congregating near the microtubule organizing center. Concomitant association of ORP1L to the Rab7–RILP complex adds an additional layer of regulation, involving a novel membrane contact site between amphisomes and the ER. Superresolution fluorescence imaging and immuno-EM revealed ORP1L on single-membrane-enclosed LC3^+^ amphisomes, juxtaposed against VAP-A on the ER, indicating an interaction occurring in *trans* rather than resulting from possible ERGIC-derived VAP-A spillover into nascent autophagosomes^54^. Formation of the amphisome-ER contact site is cholesterol dependent, advocating that via ORP1L, cholesterol can toggle decisions regarding transport of amphisomes. Why cholesterol is used to mark this step in autophagosome maturation is unclear. Possibly ORP1L confines the active transport machinery to defined regions on a vesicle, cholesterol-enriched subdomains. If so, decisions regarding transport are restricted to a few locations on a vesicle, so-called transport nodes, which potentially simplifies regulation of bidirectional transport. In support of this theory, it was recently shown that on phagosomes Rab7 and the dynein motor are recruited specifically to cholesterol-enriched subdomains to control minus-end transport^55^. Interestingly, plus-end transport of amphisomes depends on PI(3)P binding by FYCO1 (ref. 31), suggesting a broader role for lipids in the regulation of amphisome transport. ORP family members have been demonstrated to also bind phosphoinositides within their ORD domain^56^,^57^,^58^, which may also apply for the ORD domain of ORP1L. The physiological role for transport regulation by cholesterol or other putative ligands of ORP1L is unclear, but it is possible that it functions as a timer, where older amphisomes can be recognized from a distinct lipid composition of their cytosolic leaflet, leading to minus-end transport and subsequent fusion with the lysosome. On top of the transport regulating ORP1L–VAP-A contact sites, we also observed cholesterol- and VAP-A-dependent but ORP1L-independent AV/ER contact sites. These could be facilitated by other ORP family members harbouring the FFAT motif, or afforded by the divergent cholesterol-binding proteins STARD3 or STARD3NL^38^,^59^,^60^, and will be subject for further study. Thus, via ORP1L, the ER and lipids in the limiting membranes of endosomes regulate late autophagosome transport, illustrating its complexity and intercompartmental control.

In addition to their role in autophagy induction through the regulation of mTOR activity^30^,^61^, our data implicate LE/Ly as potent drivers of autophagic flux, capable of imposing restrictions on acceptance of autophagic vesicles into its fold. Fusion is thus not simply imposed by the autophagosome, but is also subject to licence by the LE. This mechanism can be exploited on the individual endosome basis and activated on a specific set of LE/Ly, for example, melanosomes or exocytic LEs/Lys, to prevent incoming cargo. Alternatively, ORP1L could serve as a quality control switch on LE/Ly to prevent incoming traffic when the endosome is not equipped to handle additional cargo, for example, just after acquiring autophagosomal cargo. Following autophagic lysosome reformation^62^, revitalized endosomes could inactivate ORP1L to take on a new degradation cycle. Given that starvation enhances AV/LE fusion^63^, it will be interesting to investigate a potential interplay between mTOR signalling and ORP1L activity.

Collectively, our data suggest (Fig. 8) that the cholesterol sensor ORP1L regulates AV/LE fusion, and attenuates recruitment of PLEKHM1 and HOPS to Rab7–RILP complexes. Rab7 effectors PLEKHM1 and RILP then jointly attract HOPS for fusion. Following fusion, the resulting amphisome utilizes Rab7–RILP-associated dynein motor machinery for minus-end-directed transport, which is also under control of the very same ORP1L, through a novel type of MCS engaging late autophagosomal structures with the ER. These data provide a molecular basis for the potential role of a major dietary lipid, cholesterol, in controlling autophagosome transport and degradation of its content in lysosomal compartments^64^,^65^.

## Methods

### Cell culture and constructs

MelJuSo cells were cultured in IMDM supplemented with 10% FCS, HeLa and HEK 293 T cells in DMEM with 10% FCS. All cells were checked for contamination by other cells (using morphological analysis and surface marker expression) and were regularly tested for mycoplasma contamination. Constructs for expression of ORP1L, Rab7, RILP, p50, VPS39, VPS41, VPS33b, VAMP8 and VAP-A were described previously^15^,^33^. HA-ORP1L and mutants were obtained by swapping GFP in the GFP-C1 vector for a 2xHA tag. ORP1LΔORDydaa was generated by mutating the residues Y477 and D478 into alanines using the primers: 5′- gaggacgagttcgctgcggcgctgtcagattccg -3′ and 5′- tctgacagcgccgcagcgaactcgtcctcgctaag -3′. Both residues were mutated because a minor interaction with VAP-A was still observed in the D478A single amino acid mutant. PLEKHM1-GFP was a kind gift from Wim van Hul (Department of Medical Genetics, University of Antwerp, Belgium)^66^ and was recloned into 2Flag-N1 using XhoI and HindIII. mRFP-mGFP-LC3 was generated by cloning mGFP and LC3 into an mRFP-C1 vector, mCherry-LC3 by cloning LC3 into an mCherry-C1 vector. All constructs were sequence verified.

### Reagents and antibodies

Lipid depletion was carried out by incubating cells in DMEM/IMDM supplemented with 5% delipidized bovine serum (Pel-Freez Biologicals) in the presence of 50 μM lovastatin (Millipore) to inhibit cholesterol production and 230 μM Mevalonate (Sigma-Aldrich) to supply essential non-sterol isoprenoids. For endosomal cholesterol accumulation, cells were treated with 3 μg ml^−1^ U-18666A (Cayman Chemicals), for lysosomal staining Lysotracker green (Molecular Probes) was used. Starvation, used for the mRFP-mGFP-LC3 experiment, was performed by culturing cells in EBSS (ThermoScientific) for the indicated time. Puromycin (Gibco) was used at 4 μg ml^−1^. Antibodies used: rabbit anti-LC3B (Novus Biologicals NB100-2220, used for all experiments except co-staining with endogenous Rab7, microscopy 1:400, western blot 1:1,000), mouse anti-LC3B (Cosmo Bio LC3-1703, only used for co-staining with Rab7, 1:100), rabbit anti-ORP1L (1:1,000), rabbit anti-GFP (1:1,000), mouse anti-CD63 (1:500) and rabbit anti-CD63 (1:100) (all described in ref. 33^33^), rabbit anti-PDI (1:10, as described in ref. 67), mouse anti-HA (Covance 16B12, 1:1,000), rat anti-HA 3F10 (Roche, 1:200), mouse anti-p62 D3 (1:500), goat anti-calnexin C-20 (1:40), goat anti-VAP-A K15 (microscopy 1:40, western blot 1:500), mouse anti-Ubiquitin P4D1 (1:50) (all Santa Cruz), rabbit anti-Rab7 D95F2 (Cell Signaling Technologies, 1:100), and mouse anti-p150^glued^ (BD Biosciences 610473, 1:100), rabbit anti-Flag F7425 and mouse anti-Flag M2 (Sigma, 1:1,000). Secondary antibodies (goat anti-mouse Alexa 405/488/568/647, goat anti-rabbit Alexa 488/568/647, donkey anti-goat Alexa 488/568, donkey anti-mouse Alexa 488/555/647 and donkey anti-rabbit Alexa 488/647) were purchased from Life Technologies and donkey anti-rat CF568 from Biotium (all 1:200).

### Transfections

For expression studies, MelJuSo and HeLa cells were transfected using Effectene (Qiagen) according to the manufacturer's instructions. Cells were transfected 1 day before fixation or lysis. For siRNA-mediated depletion, cells were reverse transfected with DharmaFECT transfection reagent #1 and 50 nM siRNA (catalogue numbers: siCtrl: D00120613-20, siORP1L: D008350-(01,05,18,19), siVAP-A: D021382-(01,02,03,04), siRILP D008787-(01,02,03,04) of the Human siGenome SMARTpool, Dharmacon) according to the manufacturer's protocol. Briefly, siRNAs and DharmaFECT were mixed and incubated for 20 min in a culture well, after which cells were added and left to adhere (reverse transfection). Three days later, cells were fixed and stained or lysed for biochemical analysis.

### Co-immunoprecipitation and western blotting

For co-immunoprecipitation experiments, cells were lysed in lysis buffer (0.5% NP-40, 5% glycerol, 150 mM NaCl, 50 mM Tris-HCl pH 8.0, 5 mM MgCl_2_ supplemented with complete EDTA-free Protease Inhibitor Cocktail (Roche)) and cleared by centrifugation. Lysates were incubated with GFP-Trap beads (Chromotek) or protein G-Sepharose 4 Fast Flow resin with the indicated antibodies and after incubation washed extensively with lysis buffer before addition of sample buffer (2% SDS, 10% glycerol, 5% β-mercaptoethanol, 60 mM Tris-HCl pH 6.8 and 0.01% bromophenol blue).

For whole cell lysate analyses, cells were lysed directly in sample buffer. Proteins were separated by SDS-PAGE (polyacrylamide gel electrophoresis) and transferred to western blot filters. Blocking of the filter and antibody incubations were done in PBS supplemented with 0.1 (v/v)% Tween and 5% (w/v) milk powder. Blots were imaged using the Odyssey Imaging System (LI-COR) or ChemiDoc (Bio-Rad). Uncropped western blot images are presented in Supplementary Fig. 8.

### cDNA synthesis and qPCR

RNA isolation, complementary DNA synthesis and quantitative PCR with reverse transcription were performed according to the manufacturer's (Roche) instructions. Signal was normalized to GAPDH and calculated using the Pfaffl formula. Primers used for detection of GAPDH and RILP were: GAPDH fw: 5′- TGTTGCCATCAATGACCCCTT -3′, GAPDH rv: 5′- CTCCACGACGTACTCAGCG -3′, RILP fw: 5′- AAGCAGCGGAAGAAGATCAA -3′, RILP rv: 5′- TTGTCATCGGAGAGCAGGAT -3′.

### Confocal microscopy

Cells were seeded on coverslips, transfected 18 h later and treated as indicated. Twenty-four hours later, cells were fixed in 3.7% formaldehyde for 10 min and permeabilized in ice-cold methanol for 2 min. Staining was performed with the antibodies mentioned above and 4,6-diamidino-2-phenylindole (Invitrogen) to stain DNA or Filipin (Cayman Chemicals) to detect cholesterol. Images were acquired using a Leica TCS SP5 confocal microscope (Leica Microsystems, Wetzlar, Germany) at × 63 magnification. For live-cell imaging, the microscope was equipped with a climate control chamber and cells were imaged for 8 min with ∼5 frames per minute. Images were quantified using Image J plugin Jacob for Mander's coefficient calculations or FIJI's Trackmate plugin for live-cell imaging and processed using Adobe Photoshop and Illustrator.

### Cryo-immunoelectron microscopy

Transfected HeLa cells were fixed for 2 h in 2% paraformaldehyde+0,2% glutaraldehyde in 0.1 M PHEM buffer (60 mM PIPES, 25 mM HEPES, 2 mM MgCl_2_, 10 mM EGTA, pH 6.9) and then processed for ultrathin cryosectioning. Briefly, 50-nm cryosections were cut at −120 °C using diamond knives in a cryoultramicrotome (Leica Aktiengesellschaft, Vienna, Austria) and transferred with a mixture of sucrose and methylcellulose onto formvar-coated copper grids. The grids were placed on 35-mm Petri dishes containing 2% gelatine. Ultrathin frozen sections were incubated at room temperature with primary antibody and then incubated with 10 nm protein A-conjugated colloidal gold (EM Lab, Utrecht University, The Netherlands) as described^68^. For double labelling, the sections were first washed and then fixed for another 10 min in 1% glutaraldehyde, blocked and incubated with the second primary antibody and 15 nm gold-labelled protein A. The sections were embedded in a mixture of methylcellulose and uranyl acetate and examined with a Philips CM10 electron microscope (FEI, Eindhoven, The Netherlands).

### Super-resolution microscopy

Super-resolution microscopy was performed with a Leica SR GSD microscope (Leica Microsystems, Wetzlar, Germany) mounted on a Sumo Stage (#11888963) for drift-free imaging. Collection of images was done with an EMCCD Andor iXon camera (Andor Technology, Belfast, UK) and an oil immersion objective (HCX PL Apo 100X, NA 1.47). Laser characteristics were 405 nm/30 mW, 488 nm/300 mW and 647 nm/500 mW, with the 405-nm laser used for back pumping and the others for wide field/TIRF imaging. Ultra clean coverslips (cleaned and washed with base and acid overnight) were used for imaging. The number of recorded frames was variable between 10,000 to 50,000, with a frame rate of 100 Hz. The data sets were analysed with the Thunder Storm analysis module^69^ and images were reconstructed with a detection threshold of 70 photons, sub pixel localization of molecules and uncertainty correction, with a pixel size of 10 nm.

### Statistical analysis and experimental set-up

All experiments shown in the paper are performed independently at least three times and images are representative of at least 10 cells viewed per individual experiment. For co-localization quantification, numerical values of individual cells were used for calculations, while for population quantifications, the averages per experiment were used. Statistical significance was calculated using an unpaired Student's *t*-test, except for normalized signals, for which a paired *t*-test was done. Statistical values are as following: *P<0.05, ^**^P<0.01, ^***^P<0.001, ^****^P<0.0001.

### Data availability

All data supporting the findings in this study are included in the article, either in the main figures or the supplementary information files.

## Additional information

**How to cite this article**: Wijdeven, R. H. *et al*. Cholesterol and ORP1L-mediated ER contact sites control autophagosome transport and fusion with the endocytic pathway. *Nat. Commun.* 7:11808 doi: 10.1038/ncomms11808 (2016).

## Supplementary Material

Supplementary Information Supplementary Figures 1-8

Supplementary Movie 1 Time-lapse imaging of MelJuSo cells transfected with mCherry-LC3 and grown under normal cell culture conditions including 8% FCS. Cells were imaged for 8 minutes with images taken every 10sec. Relates to Fig. 1.

Supplementary Movie 2 Time-lapse imaging of MelJuSo cells transfected with mCherry-LC3 and grown under cholesterol-limiting conditions. Cells were imaged for 8 minutes with images taken every 10sec. Relates to Fig. 1.

Supplementary Movie 3 Time-lapse imaging of MelJuSo cells transfected with mCherry-LC3 after exposure to U18666A to accumulate cholesterol in late endosomes. Cells were imaged for 8 minutes with images taken every 10sec. Relates to Fig. 1.

Supplementary Movie 4 3D z-stack of an autophagosome imaged by superresolution microscopy. MelJuSo cells were transfected with GFP-ORP1LΔORD (green) and stained for VAP-A (blue) and LC3 (red). Relates to Fig. 3.
