# Supplementary material for: Cholesterol and ORP1L-mediated ER contact sites control autophagosome transport and fusion with the endocytic pathway
Source: Nat Commun. 2016 Jun 10;7:11808. doi: 10.1038/ncomms11808 (PMC4906411; doi:10.1038/ncomms11808)
Supplement: Supplementary Information — Supplementary Figures 1-8 [file ncomms11808-s1.pdf]

Supplementary Information

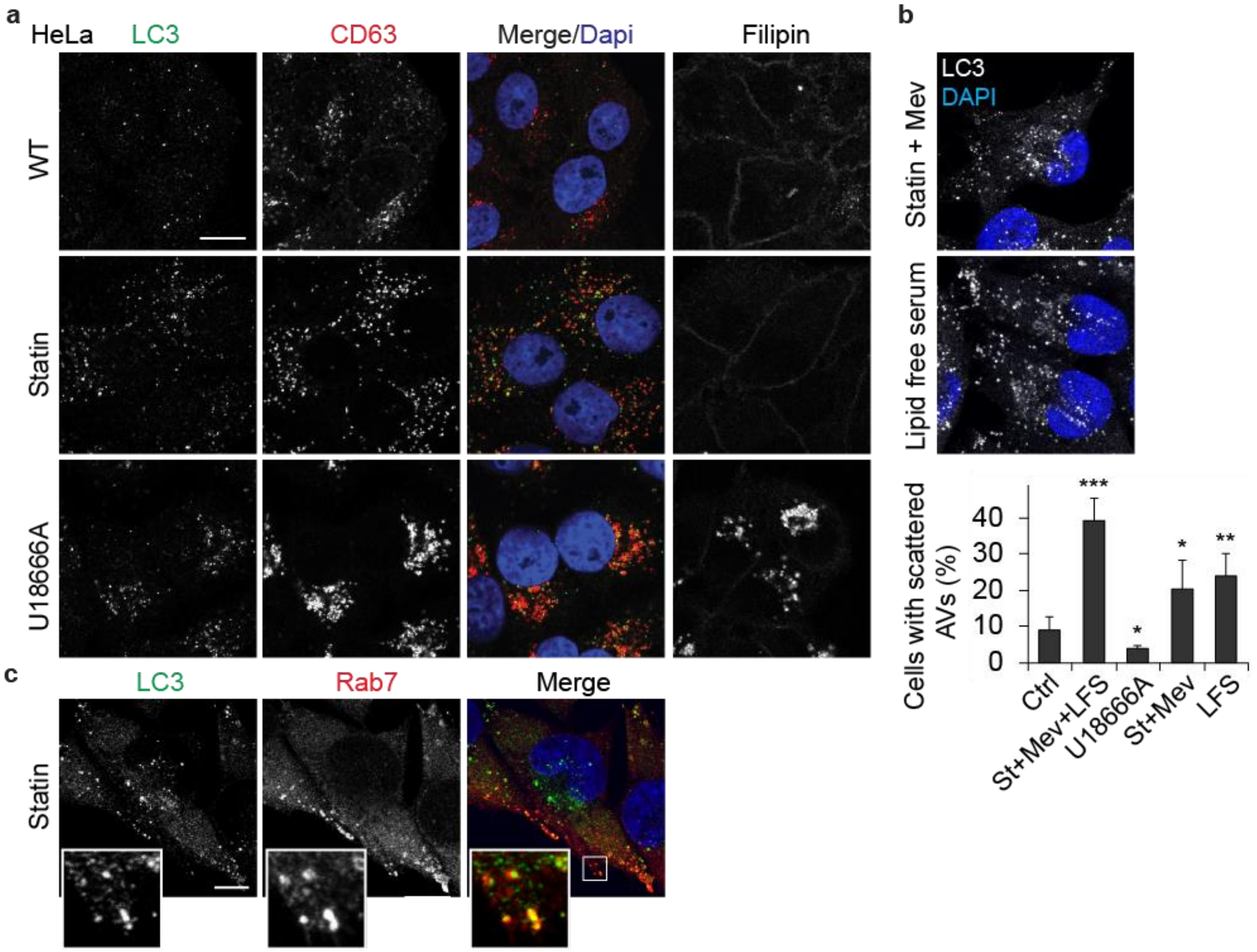

Supplementary Figure 1: cholesterol manipulation alters the positioning of autophagosomes in cells, related to figure 1. (**a**) HeLa cells were treated for 24h under conditions reducing cholesterol (50µM Lovastatin, 5% lipid free serum and 230µM Mevalonate) or with 3µM U18666A to enrich for endosomal cholesterol and stained for the indicated markers. Filipin staining was performed on different slides to verify cholesterol depletion or accumulation following the respective treatments. Bar= 10 µm. (**b**) MelJuSo cells were treated for 24h with statin and mevalonate (Statin + Mev) or lipid free serum to reduce cholesterol and stained for the indicated markers. Lower panel: quantification of the percentage of cells showing peripheral accumulation of autophagosomes, treatments as in Fig 1b and S1b. >100 cells per experiments were counted. Bars indicate mean + SD from independent triplicate experiments and means were compared using Student's t-test. (**c**) Staining of cholesterol-reduced MelJuSo cells for LC3 and Rab7. Scale bar, 10µm. (* $p < 0.05$, ** $p < 0.01$, *** $p < 0.001$).

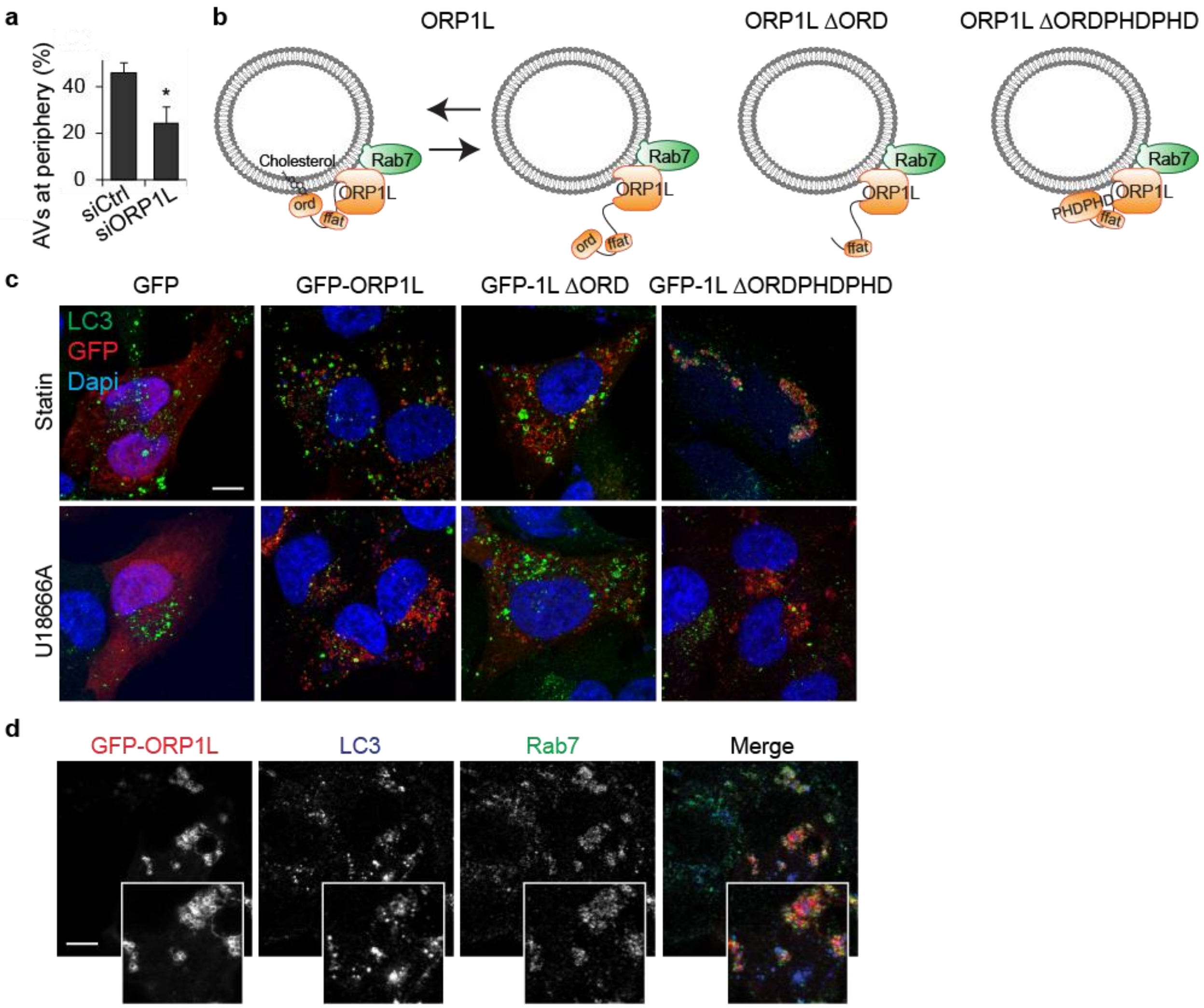

Supplementary Figure 2: ORP1L localizes to Rab7 positive autophagosomes and controls their positioning, related to figure 2. (**a**) Quantification of the number of LC3 structures in the peripheral region, from Figure 2a. Autophagosomes outside the perinuclear region, relative to the total number of LC3 vesicles per cell were quantified. Quantification on >10 cells per experiment, bars represent mean+SD of independent triplicates. Student's t-test statistical analysis. (**b**) Model of ORP1L and its mutants. ORP1L binds cholesterol from the cytosolic leaflet of endosomal membranes by its ORD domain, which covers the FFAT motif. When not interacting with the membrane (low cholesterol conditions), ORP1L (like ORP1LΔORD) has its FFAT motif exposed to interact with ER protein VAP-A. The tandem PHD domains of ORP1LΔORDPHDPHD associate to the membrane and cover its FFAT motif, mimicking the high cholesterol condition. (**c**) MelJuSo cells transfected with the indicated constructs were cultured under cholesterol manipulating conditions and stained for LC3 and DAPI. (**d**) MelJuSo cells expressing GFP-ORP1L were stained for Rab7 and LC3. Bar= 10 µm. (* $p < 0.05$).

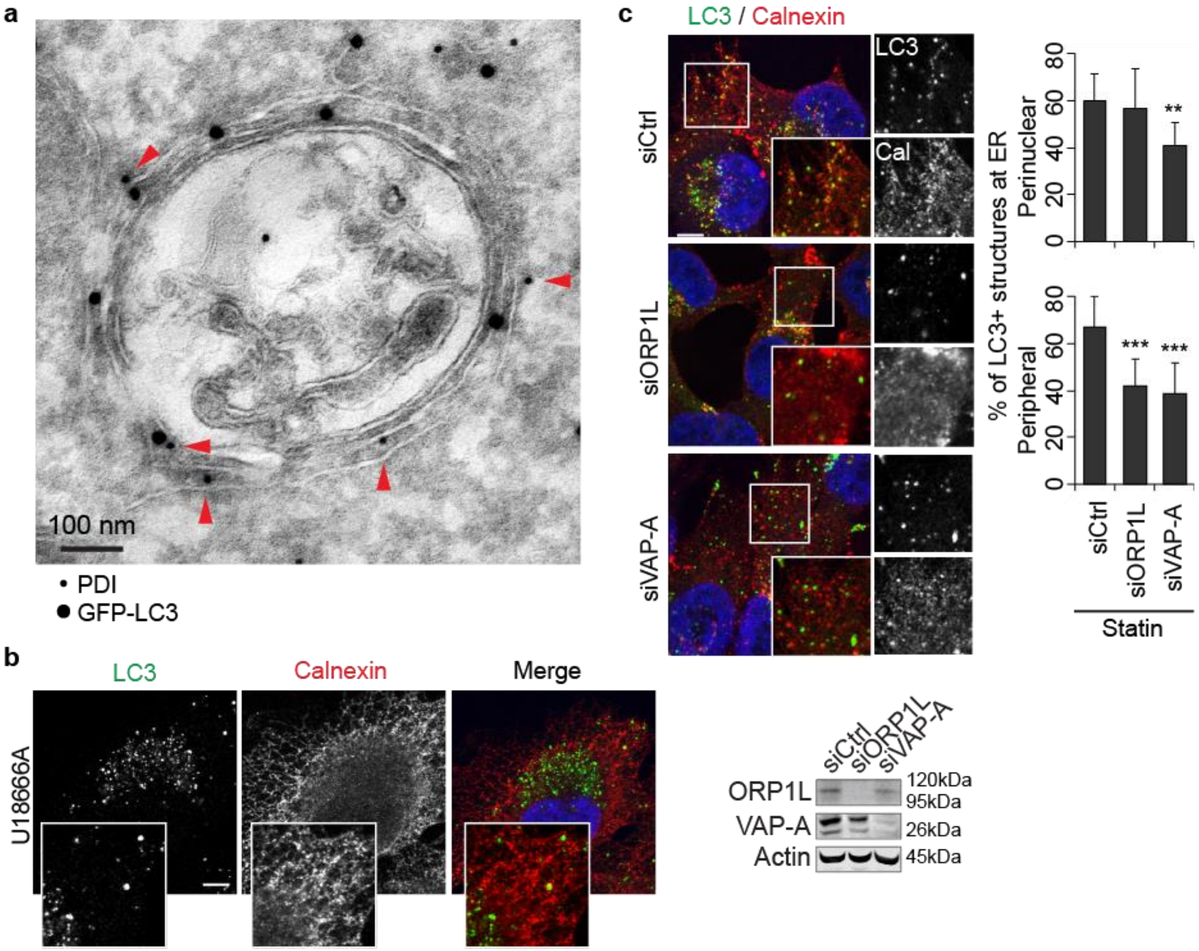

Supplementary Figure 3: ORP1L induces the formation of ER-autophagosome contact sites, related to figure 3. (**a**) Immuno-EM on HeLa cells expressing GFP-LC3 and HA-ORP1LΔORD. Cells were stained for GFP and the luminal ER marker PDI. The double membrane surrounding autophagosomes stained positive for PDI (red arrowheads), illustrating ER contact sites with autophagosomes. Bar= 100nm (**b**) MelJuSo cells enriched for cholesterol by exposure to U18666A were stained for LC3 to mark autophagosomes and Calnexin to label the ER. Bar= 10 µm.(**c**) MelJuSo cells transfected with siCtrl, siORP1L or siVAP-A were depleted for cholesterol and stained for LC3 and Calnexin. Bar= 10 µm. Fraction of peripheral or perinuclear autophagosomes co-localizing with the ER were quantified manually in >10 cells per experiment over three experiments. Bars indicate mean+SD from independent triplicate experiments. Efficient silencing of VAP-A and ORP1L was confirmed by Western blot. Molecular weight is indicated on the right. Student's t-test statistical analysis (** $p < 0.01$, *** $p < 0.001$).

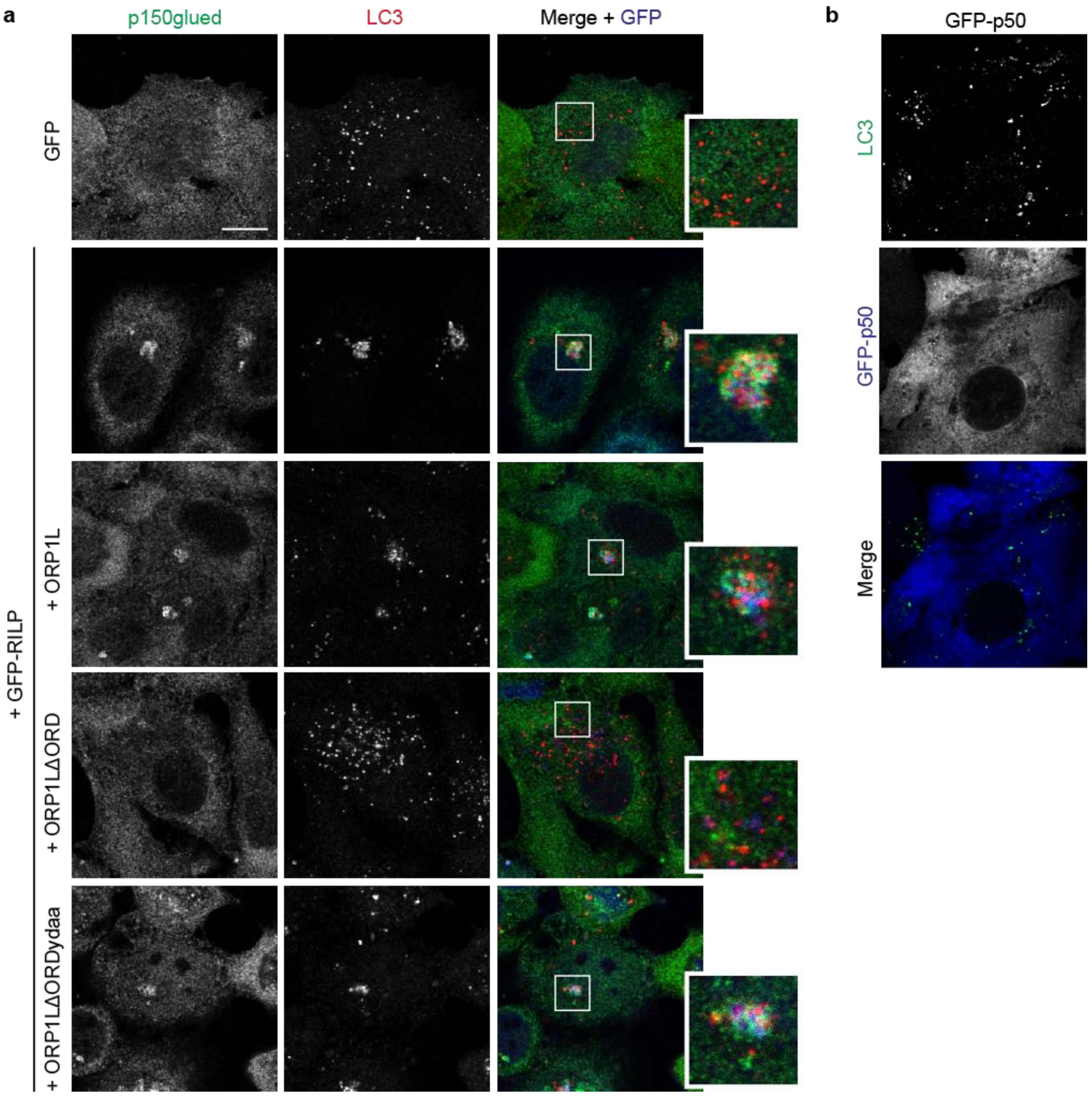

Supplementary Figure 4: RILP recruits the dynein-dynactin motor to AVs for minus-end transport, which is controlled by ORP1L, related to figure 4. (**a**) HeLa cells overexpressing GFP, GFP-RILP, or GFP-RILP and HA-tagged ORP1L constructs (as indicated) were stained for LC3 and p150^{glued}. Right: zoom-in of the indicated region. (**b**) HeLa cells transfected with GFP-P50^{dynamitin} were stained for LC3 (red). Bar= 10 µm.

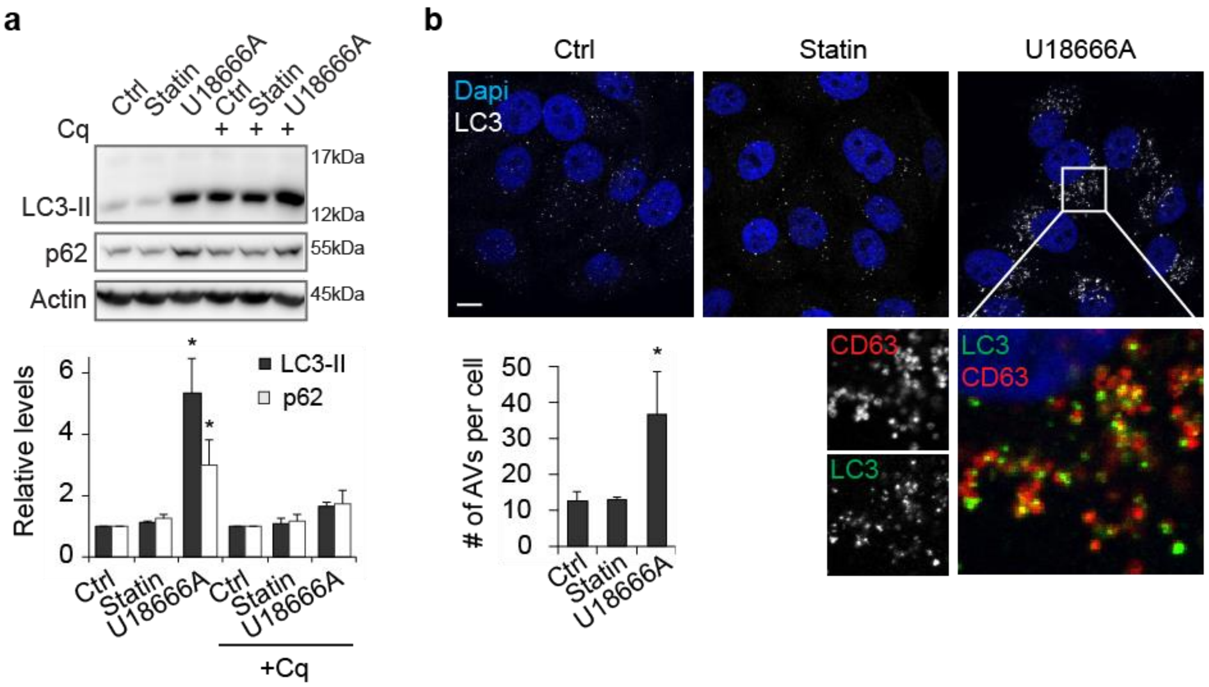

Supplementary Figure 5: effects of cholesterol manipulation on the autophagic flux. (**a**) HeLa cells were reduced (statin) or enriched (U18666A) for cholesterol for 24h and incubated for the last 4h with chloroquine (cq) when indicated. After lysis, samples were analyzed by SDS-PAGE and Western blot for the indicated proteins. Bottom: signal relative to actin was normalized to the Ctrl cells (with or without chloroquine). Bars represent mean+SD of three independent experiments, means are compared by Student's t-test. (**b**) HeLa cells were treated for 24h under conditions reducing cholesterol (50μM Lovastatin, 5% lipid free serum and 230μM Mevalonate) or with 3μM U18666A to enrich for endosomal cholesterol and stained for the indicated markers. Bar= 10 μm. Numbers of AVs per cell were quantified for >10 cells per experiment. Bars indicate mean+SD from independent triplicate experiments and significance was analyzed using Student's t-test statistical analysis. (* $p < 0.05$).

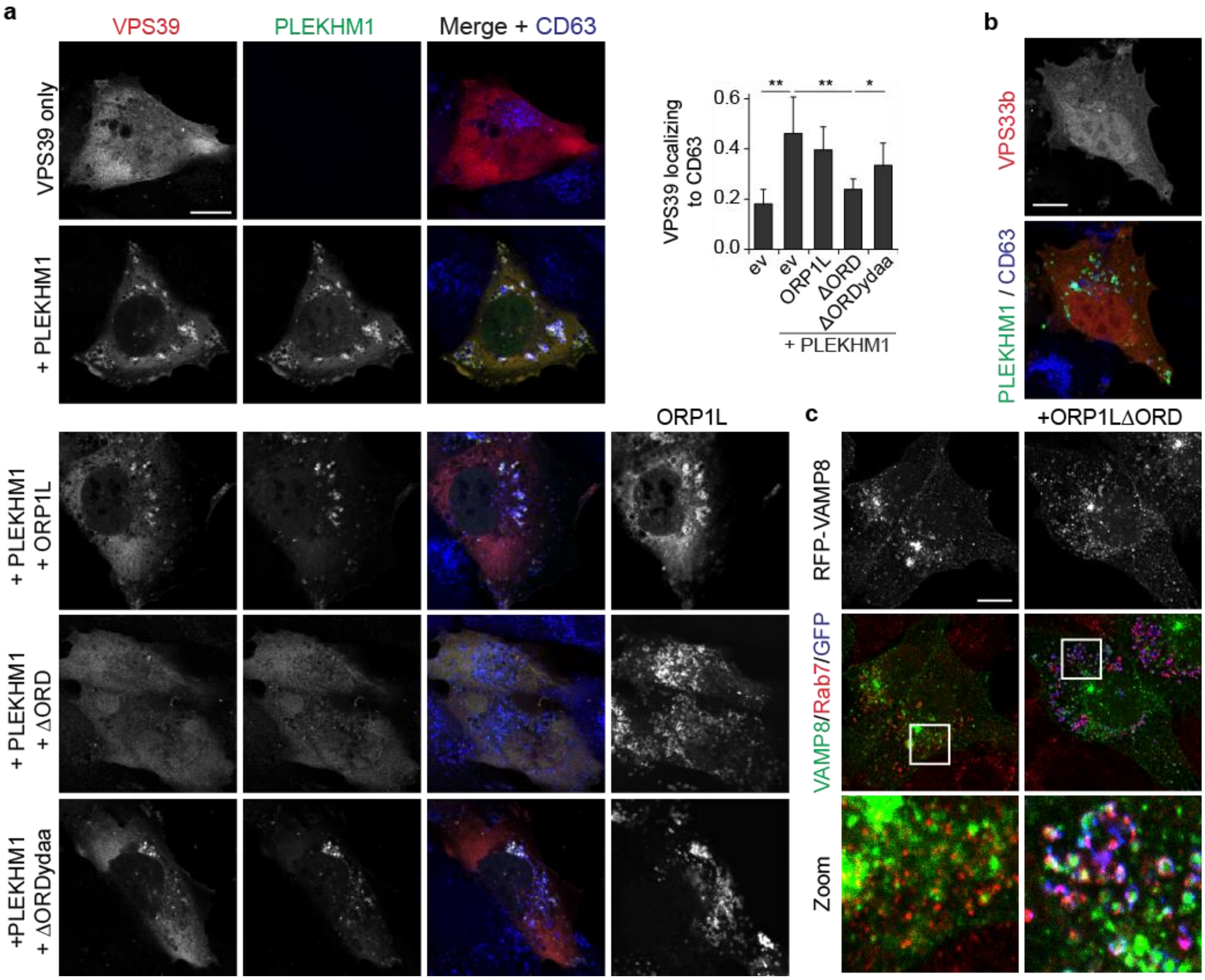

Supplementary Figure 6: ORP1L controls recruitment of PLEKHM1 and HOPS to Rab7, related to figure 6. (**a**) MelJuSo cells were transfected with GFP-tagged PLEKHM1, mRFP-VPS39 and the indicated HA-tagged ORP1L mutants and stained for CD63. Bar= 10 µm. Right: Manders coefficient for VPS39 localization to CD63 was determined for >10 cells over three independent experiments, bars represent mean+SD. Student's t-test statistical analysis. (**b**) PLEKHM1 fails to recruit VPS33b to membranes. MelJuSo cells were transfected with GFP-tagged PLEKHM1 and mRFP-VPS33b and stained for CD63. Bar= 10 µm. (**c**) ORP1L does not affect the localization of VAMP8. MelJuSo cells were transfected with either mRFP-VAMP8 or with mRFP-VAMP8 and GFP-ORP1LΔORD and stained for Rab7. Bar= 10 µm**.** Zoom-in from indicated region. ev: empty vector. (* $p < 0.05$, ** $p < 0.01$).

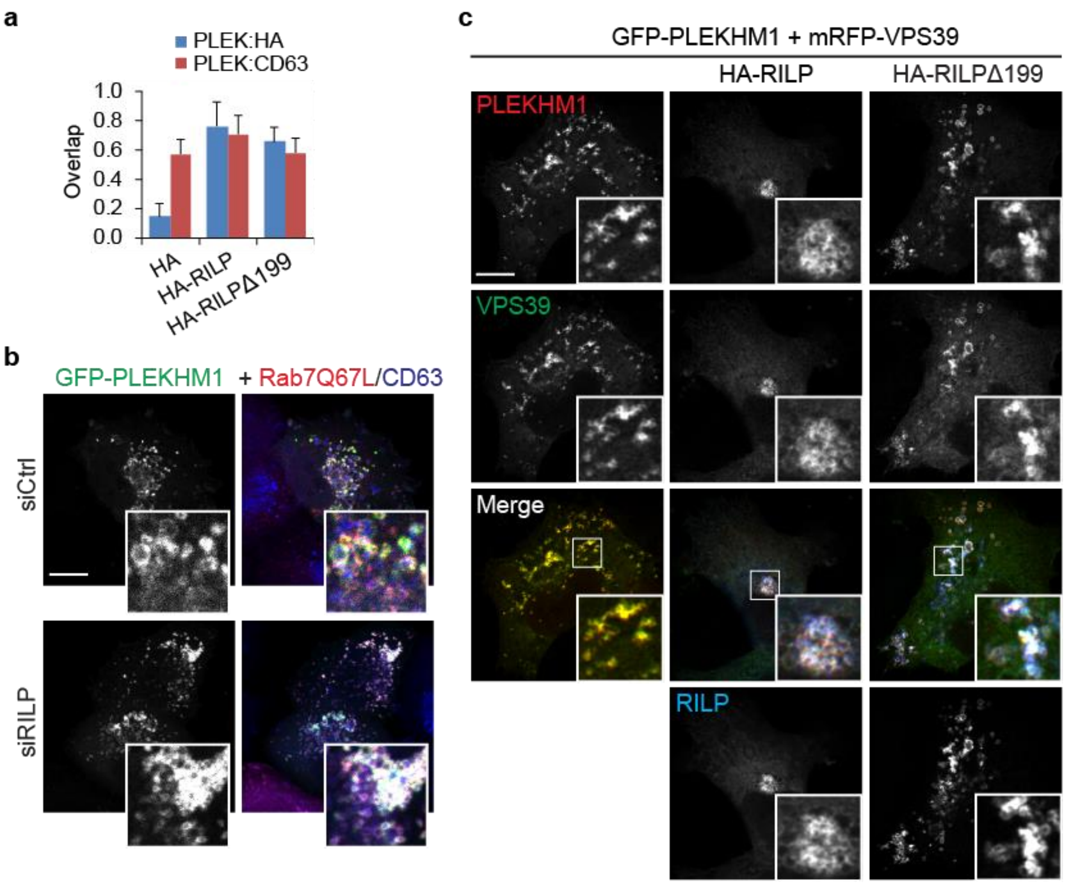

Supplementary Figure 7: RILP controls PLEKHM1 recruitment by Rab7, related to figure 7. (**a**) Quantification of Fig. 7a. Manders coefficient for PLEKHM1 localization to CD63 or HA was calculated for >10 cells over three independent experiments, bars represent mean+SD. (**b**) MelJuSo cells silenced with siCtrl or siRilp were transfected with GFP-PLEKHM1 and Myc-Rab7Q67L and stained for CD63. Bar= 10 µm. (c) MelJuSo cells were transfected with GFP-PLEKHM1 and mRFP-VPS39, together with HA-RILP or HA-RILPΔ199 when indicated. Bar= 10 µm. For all quantification experiments: bars indicate mean + SD from independent triplicate experiments.

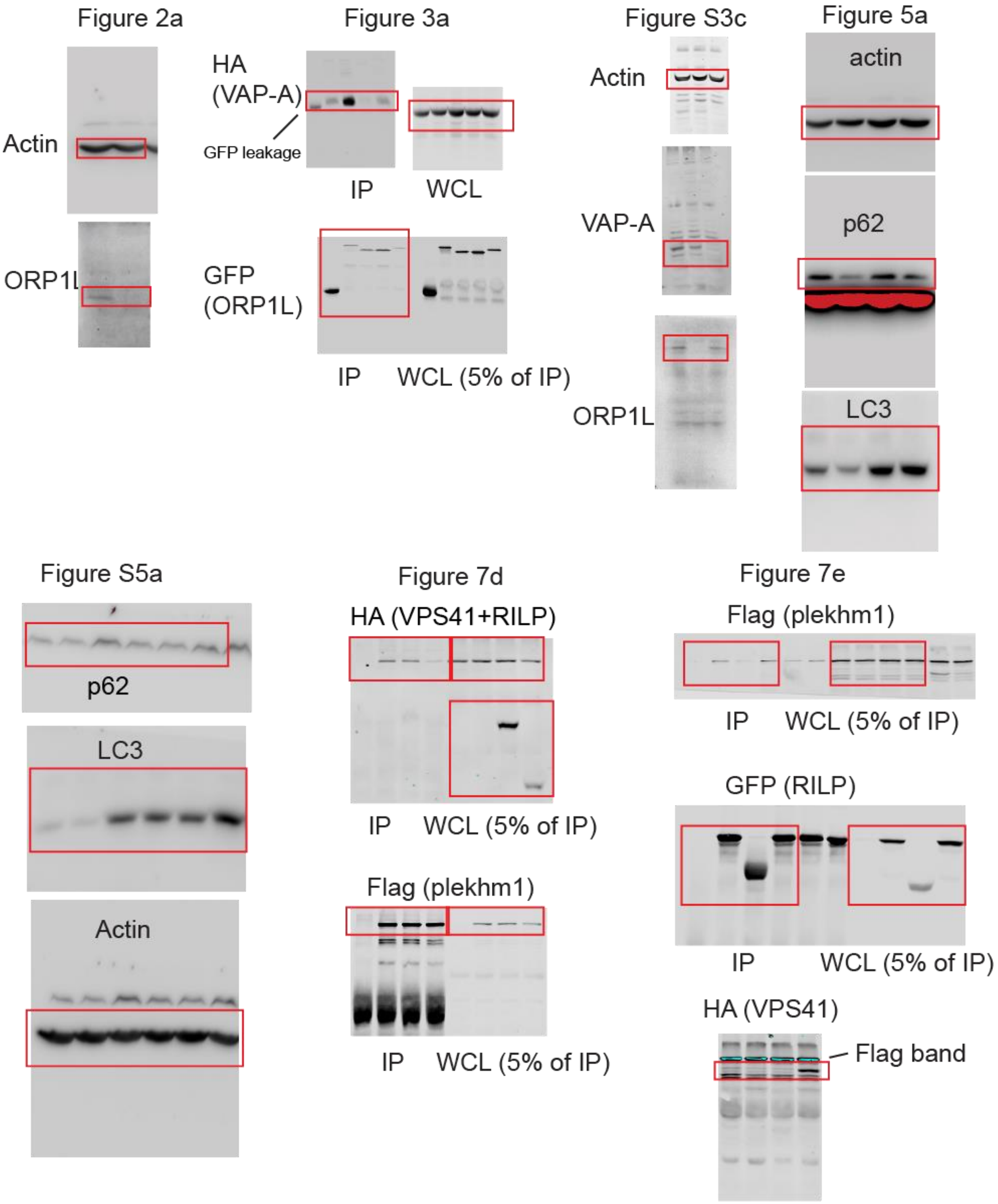

Supplementary Figure 8: Full size Western blots for all experiments.
